# Combined Experimental and Theoretical Study of the Competitive Absorption of CO_2_ and NO_2_ by a Superbase Ionic Liquid

**DOI:** 10.1021/acssuschemeng.1c01451

**Published:** 2021-05-26

**Authors:** Adam J. Greer, S. F. Rebecca Taylor, Helen Daly, Matthew G. Quesne, Nora H. de Leeuw, C. Richard A. Catlow, Johan Jacquemin, Christopher Hardacre

**Affiliations:** † School of Chemistry and Chemical Engineering, Queen’s University Belfast, David Keir Building, Stranmillis Road, Belfast BT9 5AG, Northern Ireland; ‡ Department of Chemical Engineering and Analytical Science, The University of Manchester, The Mill, Sackville Street, Manchester M13 9PL, United Kingdom; § School of Chemistry, Cardiff University, Main Building, Park Place, Cardiff CF10 3AT, United Kingdom; ∥ UK Catalysis Hub, Research Complex at Harwell, STFC Rutherford Appleton Laboratory, Didcot, Oxfordshire OX11 0FA, United Kingdom; ⊥ School of Chemistry, University of Leeds, Leeds LS2 9JT, United Kingdom; # Department of Chemistry, University College London, 20 Gordon St., London WC1H 0AJ, United Kingdom; ∇ Laboratoire PCM2E, Université de Tours, Parc de Grandmont, 37200 Tours, France; ¶ Materials Science and Nano-Engineering, Mohammed VI Polytechnic University, Lot 660-Hay Moulay Rachid, Ben Guerir 43150, Morocco

**Keywords:** ionic liquids, CO_2_ capture, NO_2_, flue gas, competitive absorption, infrared, DFT

## Abstract

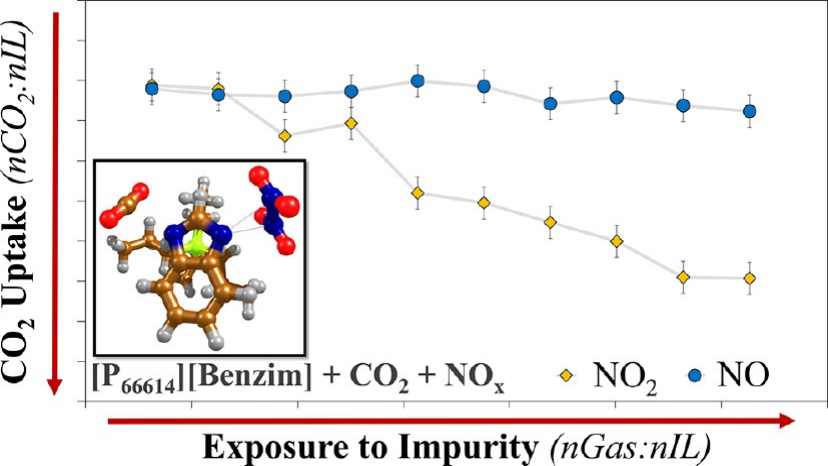

A superbase ionic liquid (IL), trihexyltetradecylphosphonium benzimidazolide ([P_66614_][Benzim]), is investigated for the capture of CO_2_ in the presence of NO_2_ impurities. The effect of the waste gas stream contaminant on the ability of the IL to absorb simultaneously CO_2_ is demonstrated using novel measurement techniques, including a mass spectrometry breakthrough method and *in situ* infrared spectroscopy. The findings show that the presence of an industrially relevant concentration of NO_2_ in a combined feed with CO_2_ has the effect of reducing the capacity of the IL to absorb CO_2_ efficiently by ∼60% after 10 absorption–desorption cycles. This finding is supported by physical property analysis (viscosity, ^1^H and ^13^C NMR, and X-ray photoelectron spectroscopy) and spectroscopic infrared characterization, in addition to density functional theory (DFT) calculations, to determine the structure of the IL-NO_2_ complex. The results are presented in comparison with another flue gas component, NO, demonstrating that the absorption of NO_2_ is more favorable, thereby hindering the ability of the IL to absorb CO_2_. Significantly, this work aids understanding of the effects that individual components of flue gas have on CO_2_ capture sorbents, through studying a contaminant that has received limited interest previously.

## Abstract 2

The mechanism of NO_2_ absorption by a superbase ionic liquid is investigated to understand the recyclability of CO_2_ capture systems.

## Introduction

Post-combustion CO_2_ capture is an important requirement of many industrial processes. The high-temperature combustion of fossil fuels produces large quantities of CO_2_ (10–15 vol %), as well as other impurities, such as SO_2_ (0.05–0.2 vol %) and NO*_x_* (0.15–0.25 vol %), which consists predominantly of NO and NO_2_.^1−3^ The reported values are obtained before any desulfurization or denitrification technologies. In particular, NO*_x_* is known to have a significant impact on health and the environment, causing the formation of atmospheric ozone and acid rain.^4^ It is therefore vital that NO*_x_* emissions are regulated (40 μg·m^–3^ a year), leading to the fitting of NO*_x_* scrubbers to power stations, comprising oxidizing and reducing agents responsible for the conversion of NO*_x_* to N_2_.^5^ Aqueous alkanolamines have been employed as CO_2_ capture sorbents, but the presence of NO*_x_* was found to result in the irreversible formation of carcinogenic nitrosamines and a decrease in CO_2_ capture efficiency.^6−8^ Ionic liquids (ILs) have also been widely investigated for the capture of CO_2_ as a non-volatile alternative to toxic alkanolamines. However, to date, the effect of NO_2_ on the ability of an IL to capture CO_2_ in a combined feed has not been investigated.

The interest in ILs stems from the ability to alter their physiochemical properties, such as thermal stability or CO_2_ absorption capacity, through changing the combination of cation and anion, which enables the tuning of their properties for specific applications.^9,10^ For example, the amount of CO_2_ absorbed by a particular IL has been shown to have a strong dependence on the anion, with conventional anions only physically absorbing small quantities of CO_2_,^11,12^ compared with task-specific ILs that incorporate amine functionality and chemically absorb up to 1 *n*CO_2_:*n*IL.^13,14^ Superbase ILs (SBILs) containing an aprotic heterocyclic anion (AHA) were developed to minimize the increase in viscosity observed in amine-functionalized ILs, and they can reversibly capture a greater than equimolar amount of CO_2_.^15−18^ Extensive studies into the absorption of other acidic gases such as SO_2_ and NO by SBILs have found that irreversible absorption was observed in many cases, often on multiple active sites within the IL, affecting the recyclability of the system.^19−25^

The effect of impurities on the CO_2_ uptake of the SBIL trihexyltetradecylphosphonium benzimidazolide, [P_66614_][Benzim], has previously been investigated in combined feeds. SO_2_ was shown to deactivate the IL through binding to the absorption site available to CO_2_, while the presence of NO exhibited little effect on the IL’s capacity for the uptake of CO_2_.^26,27^ For NO, the co-binding of CO_2_ and NO as carbamate and NONO-ate species, respectively, was observed at different N-sites of the benzimidazolide anion. However, competition for the same binding site was reported between CO_2_ and SO_2_, which markedly influenced the absorption capacity and recyclability of the IL. The differing effects of SO_2_ and NO on the absorption of CO_2_ by [P_66614_][Benzim] highlight the need to assess the components of flue gas impurities, both individually and in combination with CO_2_.

The capture of NO_2_ by ILs has been the focus of only a few studies, where it was found that an increased uptake is observed for NO_2_ compared to NO.^28−30^ To date, NO_2_ has not been studied in a combined feed with CO_2_, so its direct influence on the ability of a sorbent to capture CO_2_ is unknown. The competitive absorption of CO_2_ with industrially relevant concentrations of H_2_O, SO_2_, or NO, independently, has been investigated previously in [P_66614_][Benzim], and this IL was therefore selected for the current study to gain a comprehensive insight into more complex, multi-component feeds.^18,26,27^ The use of a recently developed analytical method utilizing mass spectrometry allows the study of this superbase IL under realistic and dry flue gas conditions, with a feed containing 14% CO_2_ and 0.2% NO_2_.^26^ Further molecular-level information was provided by density functional theory (DFT) calculations and spectroscopic data (NMR, IR, and X-ray photoelectron spectroscopy).

## Experimental Section

### Materials

Trihexyltetradecylphosphonium chloride ([P_66614_]Cl, 97.7 wt %, CAS: 258864-54-9) was procured from IoLiTec, and benzimidazole (98 wt %, CAS: 51-17-2) was purchased from Sigma–Aldrich. [P_66614_][Benzim] was prepared using a two-step synthesis method reported previously.^18^ The halide content was determined to be <5 ppm by a silver nitrate test.^31^ The water content was measured to be <0.1 wt % using a Metrohm 787 KF Titrino Karl Fischer machine. The structure and purity of the IL, after synthesis and post-absorption, were analyzed by ^1^H NMR and ^13^C NMR with a Bruker Avance II 400 MHz Ultra shield Plus and carried out as neat ILs in the presence of a glass capillary insert containing a deuterated solvent (DMSO-*d*_6_, purchased from Cambridge Isotope Laboratories Inc., CAS: 2206-27-1). Gases were obtained from BOC; argon (99.998%, CAS: 7440-37-1); carbon dioxide (99.99%, CAS: 124-38-9); nitrogen dioxide (1% in argon, CAS: 10102-44-0).

### Methods

The gas absorption measurement techniques used in this work were reported in detail previously, and the same protocol was followed in this work.^26,27^ To briefly summarize this, the uptake of a single component gas feed (1% NO_2_ in argon) by [P_66614_][Benzim] was studied gravimetrically at 22 ± 0.5 °C. A mass spectrometer-based method was utilized to study the gas phase concentrations at the outlet after the IL was exposed to a mixed gas feed of 14% CO_2_ + 0.2% NO_2_ in Ar. A series of cycles were performed consisting of a 2 h absorption period under feed conditions at 22 °C, followed by a 2 h desorption period under Ar at 90 °C.

### Analysis

The viscosity of the IL samples was measured before and after NO_2_ absorption using a TA Instruments AR2000. Elemental analysis was carried out using a Thermo Scientific Flash 2000 elemental analyzer. X-ray photoelectron spectroscopy (XPS) was performed with a Kratos AXIS Ultra DLD apparatus, with a monochromated Al Kα radiation X-ray source, charge neutralizer, and hemispherical electron energy analyzer. During data acquisition, the chamber pressure was kept below 10^–9^ mbar. The spectra were analyzed by CasaXPS and corrected for charging using the C 1s feature at 284.8 eV.

Attenuated total reflectance-infrared (ATR-IR) spectra were recorded in a modified *in situ* cell with a ZnSe crystal and a PIKE ATRMax II accessory housed in a Bruker Tensor II infrared spectrometer. A thin film of [P_66614_][Benzim] (∼250 mg) coated the ZnSe crystal in the cell before the introduction of the gas feed (14% CO_2_ in Ar, 0.2% NO_2_ in Ar, or a mixed gas feed of 14% CO_2_ with 0.2% NO_2_ in Ar) with a flow rate of 15 cm^3^·min^–1^ at 22 °C. Desorption was performed at 90 °C under Ar. The background for all spectra was the ZnSe crystal in the cell, and spectra were recorded with 8 scans at 4 cm^–1^ resolution. The spectrum of the IL before introduction of the gas feed has been subtracted from all the spectra of the IL under gas absorption.

### DFT Calculations

DFT calculations followed a similar protocol to previous work on this system, and while a brief overview will be included here, a more detailed description can be found in the literature.^27^ Calculations were performed with the Gaussian09 software package^32^ using a combination of the hybrid functional UB3LYP and the triplet-ζ basis set 6-311+G*, as reported in previous works.^33−35^ Starting geometries for [P_3333_][Benzim] models were also informed by previous molecular dynamical studies,^36^ with absorbates manually added using the ChemCraft software package.^37^ Minima structures for all possible reaction mechanisms were fully optimized without constraints with transition states located by initially running geometry scans, where only the degree of freedom connecting two minima was fixed. Full transition state optimizations were subsequently performed on the highest energy structures obtained along each reaction coordinate. The verification of both minima and transition states were carried out with the aid of analytical frequencies at 1 atm and 298.15 K, whereby only positive frequencies were observed for each minima, with each transition state possessing a single imaginary frequency for the mode associated with the reaction coordinate. Corrections for long-range interactions were included with the aid of the Grimme D3 dispersion model,^38^ while solvent effects were simulated with an implicit model of acetonitrile (ε = 35.688) using a polarizable continuum model (PCM).

## Results and Discussion

The absorption of 1% NO_2_ in Ar by [P_66614_][Benzim] was initially examined by a gravimetric technique to allow direct comparison with the literature on the uptake of individual gases. It was found that an average of 4.60 *n*NO_2_:*n*IL was absorbed at saturation (Figure S1), with the higher than equimolar capacity indicating a multi-site absorption effect for NO_2_, as was found for NO.^27^ The amount of NO_2_ absorbed far exceeds that found for 1% NO by the same IL (1.73 *n*NO:*n*IL), indicating a different mechanism of absorption for NO_2_.^27^ The regeneration of the IL at 90 °C under argon was studied (well below the IL’s decomposition temperature of 289 °C),^18^ with 3.64 *n*NO_2_:*n*IL remaining after 2 h. NO_2_ absorption is evidently not a reversible process, with the absorbed species strongly bound to the IL, which has also been observed for caprolactam-based ILs.^28^

**Figure 1:**
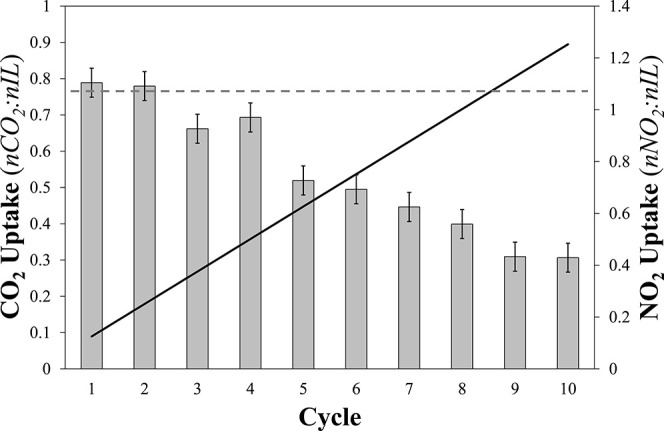
CO_2_ capacity (bars) of [P_66614_][Benzim], calculated from the MS (±0.04 *n*CO_2_:*n*IL) and calculated exposure to NO_2_ (solid line), after multiple cycles of a 2 h absorption under a feed of 14% CO_2_ and 0.2% NO_2_ in argon, and a 2 h desorption at 90 °C. A dashed line depicts the 14% CO_2_ only value (0.78 *n*CO_2_:*n*IL).

The ability of NO_2_ to compete with CO_2_ for absorption by [P_66614_][Benzim] was investigated using a combined feed through a series of absorption and desorption cycles, employing a mass spectrometry-based gas absorption rig.^26^ Realistic flue gas concentrations of 14% CO_2_ and 0.2% NO_2_ were selected, with the results shown in Figure 1 (and Table S1). It demonstrates that after two cycles in the gas absorption rig, the CO_2_ capacity of the IL was unaffected by the presence of NO_2_, with 0.78 *n*CO_2_:*n*IL still absorbed. After the third cycle, a clear decrease in CO_2_ capacity was observed, with 0.66 *n*CO_2_:*n*IL absorbed after exposure to a calculated 0.38 *n*NO_2_:*n*IL. The ability of [P_66614_][Benzim] to capture CO_2_ in the presence of NO_2_ continued to decrease, reaching 0.31 *n*CO_2_:*n*IL after 10 absorption/desorption cycles, an ∼60% reduction in capacity. It was evident that the desorption conditions (2 h under Ar at 90 °C) were unable to regenerate the original CO_2_ capacity. This strong irreversible absorption of NO_2_ is in contrast to the behavior observed with the NO co-feed, where 0.72 *n*CO_2_:*n*IL was still absorbed after 10 cycles.^27^

Following the 10 competitive absorption/desorption cycles with CO_2_ and NO_2_, the treated IL was characterized using a number of techniques. Elemental analysis showed an increase in the nitrogen content of [P_66614_][Benzim] from 6.10 to 7.67 wt % and an increase in the viscosity from 1087 to 1516 mPa·s (at 25 °C). These changes were assigned to the irreversible incorporation of nitrogen into the IL through the absorption of NO_2_. Changes in the physical properties of the IL after exposure to NO_2_ were more significant than those observed after exposure to NO (nitrogen content increased from 6.10 to 6.48% and viscosity from 1087 to 1235 mPa·s), correlating with the reduced effect of the NO impurity (0.72 *n*CO_2_:*n*IL was still absorbed after 10 cycles under NO and CO_2_).^27^ As the irreversible absorption of NO_2_ causes changes in the physical properties of the IL, NMR and XPS of the IL after exposure were performed to characterize the nature of the strongly bound NO_2_ species.

The structure was initially probed by ^1^H NMR (Figure S2), where it was expected that changes in the spectra upon the absorption of NO_2_ would be caused by a change in the environment of the protons in the heterocyclic anion, which was demonstrated by a downfield shift in the peaks at 6.34/6.91/7.36 ppm (a) to 6.57/7.10/7.65 ppm (b) after the IL was treated with 1% NO_2_ for 24 h, similar to shifts observed with SO_2_ and NO.^26,27^ The IL from the absorption rig after the final cycle (c) displayed a similar shift, showing that the same absorbed species was formed under different conditions. In both spectra after NO_2_ exposure, new peaks were observed above 13 ppm, suggesting the formation of HNO_3_ from the reaction of NO_2_ with residual water in the IL. The formation of HNO_3_ was further confirmed by IR spectroscopy of [P_66614_][Benzim] after 24 h exposure to 1% NO_2_ (Figure S4). The ^13^C NMR spectra (Figure S3) showed analogous changes, where small shifts were noted for the peaks attributed to the benzimidazolide anion (114–148 ppm) due to changes in aromaticity caused by NO_2_ absorption.

*Ex situ* XPS was further used to characterize the IL post-exposure in the absorption rig. A comparison of the N 1s region showed the presence of two new N 1s photoelectron peaks at 402.0 and 406.1 eV assigned to N–O species (Figure S5). The broad peak at 402.0 eV is assigned to absorbed NO_2_, with the peak at a higher binding energy of 406.1 eV indicating a more oxidized N species, which can be attributed to N_2_O_4_ absorption.^39,40^ Absorption of NO in the same IL resulted in a single photoelectron peak observed at 402.4 eV, which was attributed to N_2_O_2_ formation.^27,41^ These species are expected to be strongly absorbed to the IL as the high vacuum in the XPS analyzing chamber would negate the detection of any weakly absorbed gases.

ATR-IR spectra of [P_66614_][Benzim] under separate CO_2_ or NO feeds have been reported previously, showing characteristic bands for the absorbed species, with additional changes in the spectra observed due to changes in the aromaticity of the [Benzim]^−^ anion.^27^ For CO_2_, reversible absorption and an equimolar capacity for absorption presented a simpler system than that for multi-site absorption, which has been reported here for NO_2_ and NO.^27^ With the higher absorption capacity of [P_66614_][Benzim] for NO_2_ and the formation of a strongly bound species, a combined approach by theoretical DFT calculations and FTIR was utilized to investigate the nature of the absorbed species in [P_66614_][Benzim].

DFT calculations revealed that the formation of an N_2_O_4_ dimer, physically absorbed to the [Benzim]^−^ anion, was the most thermodynamically favored absorption mode (Table 1a). The charge perturbation from the [Benzim]^−^ anion strongly enhanced the rate of dimerization of NO_2_, pushing the equilibrium towards N_2_O_4_ formation by strongly absorbing the dimer, thus removing it from the gas phase. The N_2_O_4_ dimer was found to physisorb strongly to the [Benzim]^−^ anion, with a zero-point corrected absorption enthalpy (*E*_ZPE_) of −107.8 kJ·mol^–1^ (Tables S2 and S3). Although the absorption of N_2_O_4_ is energetically very favorable, the absorbed geometry together with a detailed comparison of grouped Mulliken charges (Tables S4 and S5) indicates that it is a purely physical process without charge transfer from the anion, in comparison to that of chemically absorbed CO_2_. Instead, there are extremely strong dipole interactions between the two δ^+^ nitrogen centers of the absorbate and the formal negative charge on the anion.

**Table 1:**
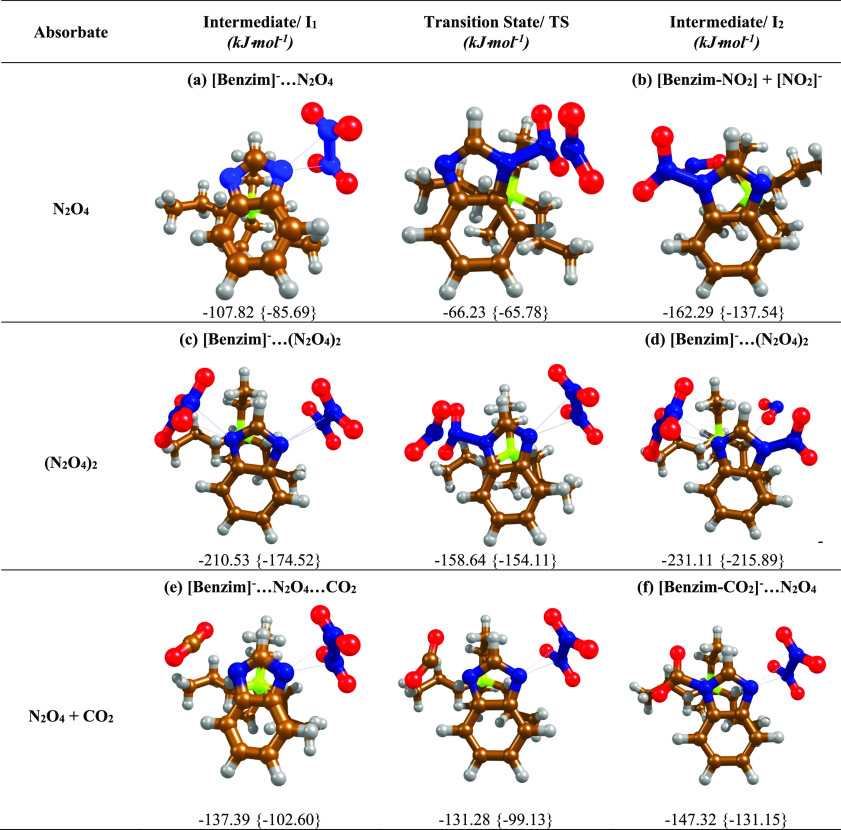
Reaction Landscapes Showing Intermediates (I) and Transition States (TS) for N_2_O_4_ and N_2_O_4_/CO_2_ Absorption by [P_3333_][Benzim], Depicting Potential Energy Surfaces for (a) Absorption of N_2_O_4_ and (b) Subsequent Heterolytic Cleavage of the N–N Bond, (c) Absorption of Two Moles of N_2_O_4_ and (d) the Barrier to Cleavage of the N–N Bond, and (e,f) the Absorption of CO_2_ by [Benzim-N_2_O_4_]^a^

a Values are given in kJ·mol^–1^ with zero-point corrected gas phase and {solvent} corrected energies calculated at B3LYP/6-311+G* level of theory (pseudo bonds = physisorption, solid bonds = chemisorption).

Furthermore, the physical absorption of two N_2_O_4_ dimers at both N-sites of the [Benzim]^−^ anion was energetically favorable, with an absorption enthalpy of −210.5 kJ·mol^–1^ (Table 1c). The multi-site absorption and greater than equimolar absorption of NO_2_ to the IL correlates with the large gravimetric uptake capacity (4.60 *n*NO_2_:*n*IL), and the high absorption enthalpies relate to the drop in CO_2_ capacity observed. These findings are also consistent with the experimentally observed results, where only a small decrease in absorbed NO_2_ was observed after desorption, probably due to the loss of weaker, physically bound, NO_2_.

**Figure 2:**
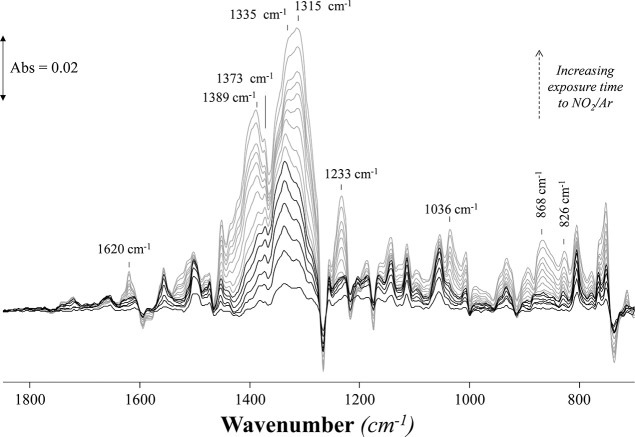
ATR-IR spectra of [P_66614_][Benzim] exposed to a feed of 0.2% NO_2_ in Ar from 0 to 2 min. The spectrum of the IL before introduction of NO_2_ has been subtracted from all spectra recorded under the NO_2_ feed. Studied at 22 °C with a flow rate of 15 cm^3^·min^–1^.

ATR-IR spectra recorded during the absorption of 0.2% NO_2_ in Ar by [P_66614_][Benzim] are shown in Figure 2. It is evident that upon the introduction of NO_2_, a series of overlapping bands in the 1300–1400 cm^–1^ region increased in intensity. Interestingly, upon initial exposure to the feed, a band at 1335 cm^–1^ was the most intense in this region, but with increasing exposure time, a band at 1315 cm^–1^ dominated the spectra. In addition, bands at 1620, 1233, and 1036 cm^–1^ increased in intensity with exposure to the NO_2_ feed. These changing bands could indicate a change in the nature of the absorbed species or absorption of a NO_2_ species at another N-site on the [Benzim]^−^ anion, as was observed with NO.^27^

Calculated vibrational spectra for one or two moles of physisorbed N_2_O_4_ to N-sites on the [Benzim]^−^ anion showed indistinguishable spectral profiles, with theoretically derived bands at 1426, 1308, and 847 cm^–1^ for one mole of physically absorbed N_2_O_4_ (Table 2) and 1433–1427, 1313–1305, and 847 cm^–1^ for two moles of N_2_O_4_. The bands that formed upon the introduction of NO_2_ at 868, 1335 and 1373 cm^–1^ correspond with the calculated bands for physisorbed N_2_O_4_ (Table 2). Bands at 1764/68 and 1825/24 cm^–1^ were predicted for the v_asym_(O–N–O) vibration when one/two moles of N_2_O_4_ were physically absorbed, but these bands were not observed experimentally, as described by the other authors.^42−44^

**Table 2:**
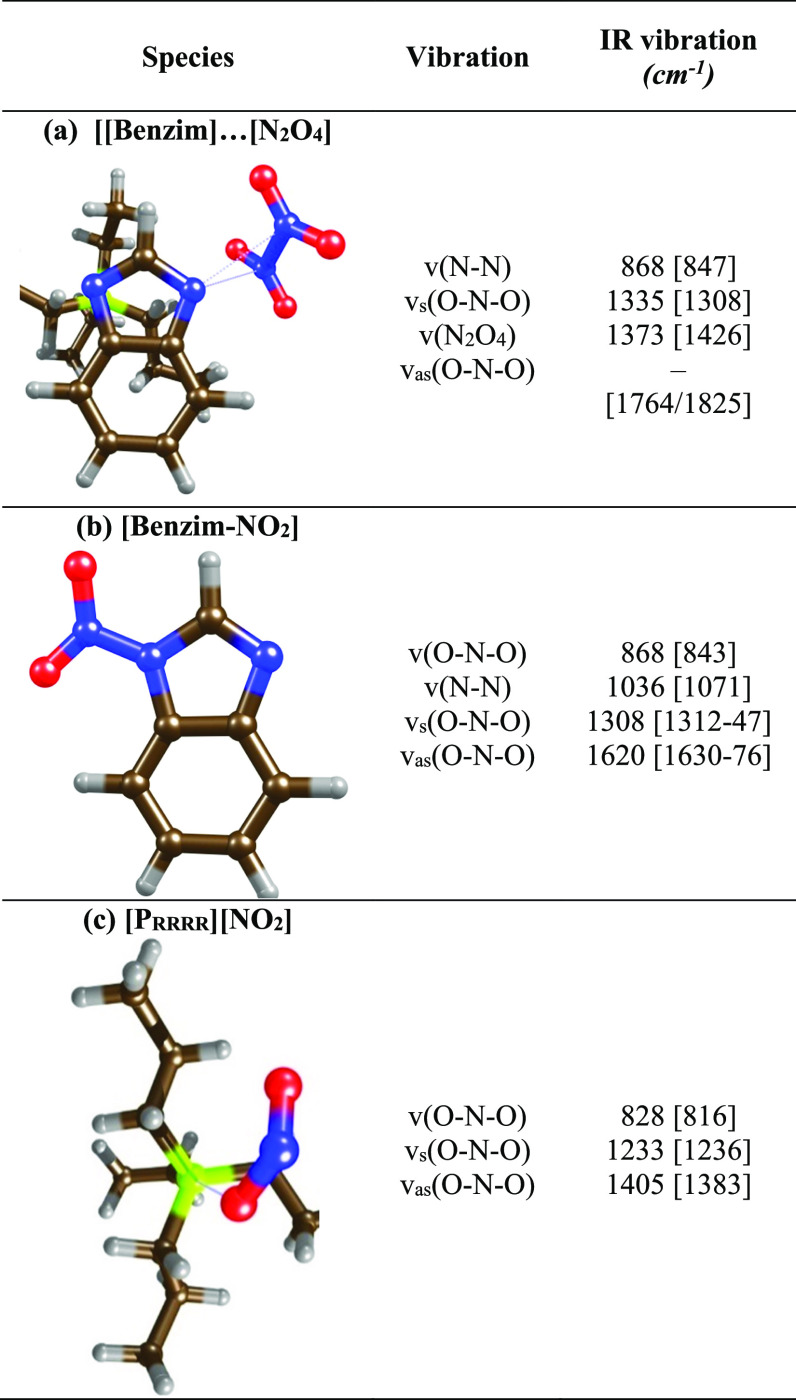
Depicts the Experimental and [Theoretical] IR Vibrations when [P_66614_][Benzim] Is Exposed to NO_2_

While physisorbed N_2_O_4_ was observed initially (eq 1), the spectra showed changes with time on stream, which were not simply an increase in the intensity of the bands assigned to N_2_O_4_, thus suggesting the evolution of the absorbed species with an increasing NO_2_ concentration. The N–N bond of absorbed N_2_O_4_ has been reported to undergo heterolytic cleavage, forming [NO_3_]^−^ and [NO]^+^ ionic species due to perturbation by an external charged species (eq 2 and 3).^45,46^ The energy diagrams in Table 1 show the most favored reaction pathway following the physisorption of one and two moles of N_2_O_4_. In these reaction schemes, the heterolytic cleavage of the N–N bond of absorbed N_2_O_4_ is favorable (b), but the activation barrier for the disproportionation reaction to NO^+^/NO_3_^–^ was above 100 kJ mol^–1^ and was considered too high (Figure S6). The cleavage of the N–N bond to form [NO_2_]^+^ and [NO_2_]^−^, however, can occur owing to the lower barrier (41.6 kJ·mol^–1^), forming a thermodynamically stable complex, which is the favored theoretical pathway following the initial physisorption of N_2_O_4_ on [P_66614_][Benzim].
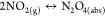
1
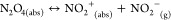
2
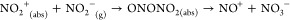
3

The theoretical pathway involving [NO_2_]^+^ and [NO_2_]^−^ species has significant implications for the absorption capacity of the IL. The [NO_2_]^+^ ion is proposed to absorb chemically to the [Benzim]^−^ anion, leading to a neutral [Benzim-NO_2_] complex (Table 2b). The neutralization of the [Benzim]^−^ anion removes the active site for absorption, causing deactivation of the anion/IL, and provides evidence for the decreased CO_2_ capacity. The [NO_2_]^−^ ion stabilizes the positively charged [P_3333_]^+^ cation by orienting the negatively charged oxygen atoms into a position to counter the positively charged phosphorus (Table 2c). It could be proposed that the [NO_2_]^−^ ion reacts with, or deprotonates, the acidic α-protons close to the phosphonium cation center (P–CH_2_−), but this pathway was not observed (see Table 2c, which shows the lowest energy structure).^47^ Additionally, Table 1c,d shows that when two moles of N_2_O_4_ are physically bound, which is more likely at increased exposure times, the heterolytic cleavage of the N–N bonds has little driving force due to similar initial and final state energies, as well as a higher barrier (51.9 kJ·mol^–1^). Thus, the formation of the neutral [Benzim-NO_2_] complex (Table 1d) is expected to be equilibrium-limited, leading to the gradual decrease in the CO_2_ capacity of the IL.

**Figure 3:**
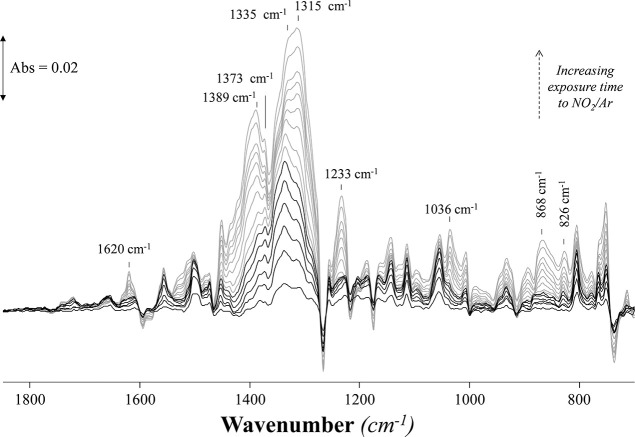
ATR-IR spectra of [P_66614_][Benzim] exposed to a feed of (i) 0.2% NO_2_ in Ar after (a) 1 min and (b) between 1 and 5 min with the subtraction of the spectrum recorded at 1 min; and (ii) 14% CO_2_ + 0.2% NO_2_ in Ar for 0–2 min. Studied at 22 °C with a flow rate of 15 cm^3^·min^–1^. The color of the labeled bands indicates physisorbed N_2_O_4_ (black), changes in the aromaticity of the IL (gray), chemical absorption of CO_2_ (green), [Benzim-NO_2_] (blue), and [P_66614_][NO_2_] (red).

To probe the evolution of the bands in Figure 2, the spectrum after 1 min under the NO_2_ feed was subtracted from all subsequent spectra (Figure 3) to allow comparison with the theoretically determined band positions of the [Benzim-NO_2_] and [P_RRRR_][NO_2_] complexes (Table 2). A new band was observed at 1233 cm^–1^, attributed to the formation of a [NO_2_]^−^ anion, and related to vibrations at 1405 and 828 cm^–1^. Evidence of a new N–N bond was observed at 1036 cm^–1^, from the formation of the neutral [Benzim-NO_2_] complex, associated with bands at 1620, 1308, and 868 cm^–1.^^48−50^ The DFT calculated species correlated well with the observed ATR spectra, and additional features were due to changes in the aromaticity of the [Benzim]^−^ anion, as observed with CO_2_/SO_2_/NO.^26,27^ Interestingly, the changes in the anion aromaticity are observed during the physical absorption of N_2_O_4_, showing that the strong initial interaction distorts the charge density of the anion.

Combining both the DFT calculations and the FTIR spectroscopic study allowed the determination of the absorbed species and how the speciation changed with time. The DFT results indicate the thermodynamically stable intermediates, while FTIR provides evidence for the proposed intermediates and the kinetics of absorption. Together, the results indicate the strong physical absorption of N_2_O_4_ followed by the deactivation of the IL through the formation of [Benzim-NO_2_]/[P_RRRR_][NO_2_] complexes at increased exposure times, which would be expected to result in a loss of absorption sites for CO_2_ and the co-absorption of CO_2_ was therefore studied as well.

A co-feed of 14% CO_2_ and 0.2% NO_2_ in Ar was investigated, simulating conditions in the gas absorption rig (Figure 3ii), to study whether bands assigned to the neutral complex would form and influence the CO_2_ absorption capacity of the IL (as observed in the absorption rig results). The chemical absorption of CO_2_ results in the formation of a carbamate species, with bands at 1709 (C=O) and 1281 cm^–1^ (N–COO^–^) quickly increasing during the first minute of exposure to the feed, which is caused by the greater concentration of CO_2_ in the feed.^26^ Simultaneously, bands associated with the physical absorption of N_2_O_4_ grow (1373 and 1334 cm^–1^) due to the strong absorption enthalpy of the species (−107.8 kJ·mol^–1^) (Table 1a). As the exposure time increases, the band at 1709 cm^–1^ (C=O) is noted to decrease, while weak bands at 1233, 1036, 868, and 828 cm^–1^ appear, indicating the chemical absorption of NO_2_^+^ to the [Benzim]^−^ anion and the formation of [P_66614_][NO_2_]. This correlates with the stepped reduction in CO_2_ capacity observed in the gas absorption rig. Subtracted data in Figure S7 show that bands due to the heterolytic cleavage of physisorbed N_2_O_4_ are observed to form with and without the presence of CO_2_. The absorption of CO_2_ does not hinder the absorption and subsequent heterolytic cleavage of N_2_O_4_, which, however, deactivates the IL to further absorption of CO_2_.

The physical absorption of N_2_O_4_ at one site drastically changes the kinetics and thermodynamics of absorption at the remaining site, where the barrier for CO_2_ activation is low (6.1 kJ·mol^–1^) (Table 1e,f). Calculations show that the co-absorption of N_2_O_4_ and CO_2_ to separate N-sites is thermodynamically stable (−147.3 kJ·mol^–1^), aided by the low concentration of NO_2_. After an extended period, both the deactivation of the [Benzim]^−^ anion and the larger absorption enthalpy of the binding of two moles of N_2_O_4_ (−210.5 kJ·mol^–1^) suggest that decreases in CO_2_ capacity are observed due to the loss of absorption sites. The reaction mechanism between the IL and CO_2_/NO_2_ is depicted in Figure S9, showing how the binding of a second N_2_O_4_ dimer further deactivates [P_66614_][Benzim] for CO_2_ capture, before the formation of the [Benzim-NO_2_]/[P_RRRR_][NO_2_] complexes occurs at increased exposure times. The irreversible nature of the bound species is further demonstrated through studying the regeneration of the IL at 90 °C (Figure S8), where it was clear that the NO*_x_* species could not be fully desorbed from [P_66614_][Benzim].

These results differ from those when NO is in the co-feed, where the co-absorption of both CO_2_ and NO was observed.^27^ The results obtained previously with NO were rationalized by the reaction landscape depicted in Figure S10, where the reduction of CO_2_ is a barrierless process, and the activation barrier to form the NONO-ate complex is relatively high, showing a preference for the absorption of CO_2_. After the formation of the carbamate, slow deactivation of the IL is observed in the presence of NO *via* a CO_2_ bond intermediate (which enables NO to co-bind). Using *in silico* techniques, this observation was explained by the relatively high kinetic barriers for the formation of the NO*_x_* species bound to the IL from NO, as opposed to NO_2_. Therefore, it is evident that an alternative absorption mechanism occurs, accelerating the effect that NO_2_ has on the uptake of CO_2_.

**Figure 4:**
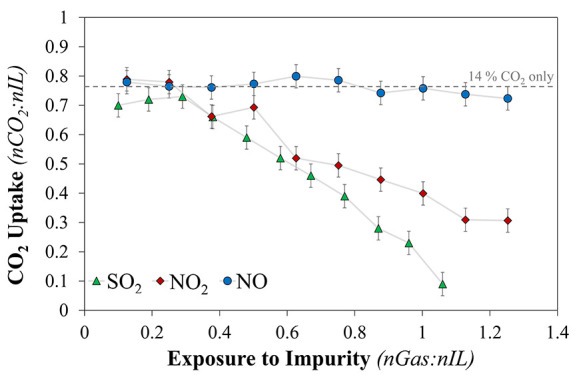
CO_2_ uptake of [P_66614_][Benzim] after exposure to an increasing amount of flue gas impurity after multiple absorption/desorption cycles of a feed containing 14% CO_2_ and 0.2% impurity; dashed line, 14% CO_2_ only value. Green triangles, SO_2_. Red solid diamonds, NO_2_. Blue circles, NO. Adapted with permission from ref (26). Copyright 2018 American Chemical Society. Adapted with permission from ref (27). Copyright 2019 American Chemical Society

A summary of the effect of the different waste gas stream impurities on [P_66614_][Benzim] studied here is shown in Figure 4. It is clear that SO_2_ has the greatest effect on reducing the CO_2_ uptake in ILs followed by NO_2_ and finally NO, in clear accord with the calculated absorption energies of −123.9,^27^ −107.8, and −91.1 kJ·mol^–1^, respectively (CO_2_ = −52.1 kJ·mol^–1^). It should be noted that, whereas the formation of the NONO-ate is thermodynamically stable (from the absorption of NO), this process is kinetically slow, hindered by the high concentration of CO_2_ in the gas feed.

## Summary and Conclusions

The accelerated effect of a flue gas contaminant, NO_2_, on the deactivation of [P_66614_][Benzim] was investigated and compared to NO. Industrially relevant gas concentrations were used to demonstrate how small amounts of impurities can dramatically change the capability of a sorbent to capture CO_2_, and in this particular case, an ∼60% decrease in CO_2_ capacity was observed. This decrease was corroborated by other experimental techniques and DFT calculations, showing the importance of considering contaminants when designing ionic liquids, or new sorbents in general, for CO_2_ capture. Spectroscopic results in combination with DFT calculations showed that NO_2_ was predominately strongly physically absorbed on multiple sites in the form of N_2_O_4_. This species was then found to undergo heterolytic cleavage resulting in the deactivation of the IL. The ability to investigate the effects of flue gas impurities, individually and competitively, on the uptake of CO_2_, is an important experimental tool in the development of new sorbents and highlights the need for rigorous experimental methods, preferably in tandem with theoretical studies and the investigation of more complex, realistic multi-component feeds.

Overall, these results show the importance of investigating the effect of flue gas contaminants. Further studies into the feasibility and optimization of ILs in such a process are still required, but a consideration of impurities and sorbent recyclability is an essential factor. Significant improvements in tuning the selectivity and absorption enthalpies of ILs to reversibly capture NO*_x_* or SO_2_ at high efficiencies are challenging, and alternative methods of removing acidic impurities will still be required (gas scrubbers and traps). Additionally, tuning the basicity of the IL by selecting anions with different p*K*_a_ values presents an interesting opportunity for the pretreatment of waste gas feeds, where contaminants such as NO*_x_* could be reversibly captured.

## Supporting Information Available

Gravimetric results (Figure S1); absorption rig data displayed in Figure 1 (Table S1); NMR data and ^1^H/^13^C NMR spectra (Figures S2 and S3, respectively); ATR-IR spectra after the absorption of 1% NO_2_ (Figure S4); N 1 s XPS spectra (Figure S5); calculated absorption energies (Tables S2 and S3) and grouped Mulliken charges/spin populations for various gases to [P_3333_][Benzim] (Tables S4 and S5); potential energy landscape of oxygen transfer from bound NO_2_^+^ (Figure S6); ATR-IR difference spectra of the absorption of NO_2_ and NO_2_ + CO_2_ co-feed (Figure S7); ATR-IR spectra showing desorption of the NO_2_, and NO_2_ + CO_2_ co-feed (Figure S8); mechanism depicting the effect of N_2_O_4_ adsorption on the CO_2_ recyclability of [P_66614_][Benzim] (Figure S9); potential energy landscape of CO_2_/NO absorption (Figure S10); listed Cartesian co-ordinates (PDF)
