# Supplementary material for: Combined Experimental and Theoretical Study of the Competitive Absorption of CO2 and NO2 by a Superbase Ionic Liquid
Source: ACS Sustain Chem Eng. 2021 May 26;9(22):7578–86. doi: 10.1021/acssuschemeng.1c01451 (PMC8296676; doi:10.1021/acssuschemeng.1c01451)
Supplement: Supplementary file 1 — sc1c01451_si_001.pdf [file sc1c01451_si_001.pdf]

# Supporting Information

# Combined Experimental and Theoretical Study of the Competitive Absorption of $CO_2$ and $NO_2$ by a Superbase Ionic Liquid

*Adam J. Greer*[†,‡,]*, *S. F. Rebecca Taylor*[‡], *Helen Daly*[‡], *Matthew G. Quesne*[#,⸸], *Nora H. de Leeuw*[#,∵], *C. Richard A. Catlow*[#,⸸], *Johan Jacquemin*[§,ν,]*, *Christopher Hardacre*[‡,]*

[†] School of Chemistry and Chemical Engineering, Queen's University Belfast, David Keir Building, Stranmillis Road, Belfast, BT9 5AG, Northern Ireland

[‡] Department of Chemical Engineering and Analytical Science, The University of Manchester, The Mill, Sackville Street, Manchester, M13 9PL, United Kingdom

[#] School of Chemistry, Cardiff University, Main Building, Park Place, Cardiff, CF10 3AT, United Kingdom

[⸸] UK Catalysis Hub, Research Complex at Harwell, STFC Rutherford Appleton Laboratory, Didcot, Oxfordshire, OX11 0FA, United Kingdom

[∵] School of Chemistry, University of Leeds, Leeds, LS2 9JT, United Kingdom

[§] Université de Tours, Laboratoire PCM2E, Parc de Grandmont, 37200, Tours, France

[v] Materials Science and Nano-Engineering, Mohammed VI Polytechnic University, Lot 660-

Hay Moulay Rachid, Ben Guerir 43150, Morocco

**AUTHOR INFORMATION**

Co-Corresponding Authors:

* Christopher Hardacre, Tel: +44 (0) 161 3062672, E-mail: c.hardacre@manchester.ac.uk

* Johan Jacquemin, Tel: +33 (0) 2 47 36 73 29, Fax: +33 (0) 2 47 36 70 73, E-mail: jj@univ-tours.fr

* Adam Greer, Tel: +44 (0) 161 3062227, E-mail: adam.greer@manchester.ac.uk

# SI Table of Contents (22 pages, 9 Figures, 5 Tables)


| *Page* | *Item* |
|---|---|
| S3 | Figure S1. Gravimetric results. |
| S4 | Table S1. Absorption rig data displayed in Figure 1. |
| S6 | Figure S2. $^{1}$H NMR spectra. |
| S7 | Figure S3. $^{13}$C NMR spectra. |
| S8 | Figure S4. ATR-IR spectra after the absorption of 1% $NO_2$. |
| S9 | Figure S5. N 1s XPS spectra. |
| S10 | Tables S2-S3. Calculated absorption energies. |
| S11 | Tables S4-S5. Grouped Mulliken charges/spin populations for various gases to [$P_{3333}$][Benzim]. |
| S12 | Figure S6. Potential energy landscape of oxygen transfer from bound $NO_2^+$. |
| S13 | Figure S7. ATR-IR difference spectra of the absorption of $NO_2$ and $NO_2$ + $CO_2$ co-feed. |
| S14 | Figure S8. ATR-IR spectra showing desorption of the $NO_2$, and $NO_2$ + $CO_2$ co-feed. |
| S15 | Figure S9. Proposed mechanism showing the effect of $N_2O_4$ absorption on the $CO_2$ recyclability of [$P_{66614}$][Benzim]. |
| S16 | Figure S10. Potential energy landscape of CO2/NO absorption. |
| S17 | Listed Cartesian co-ordinates. |

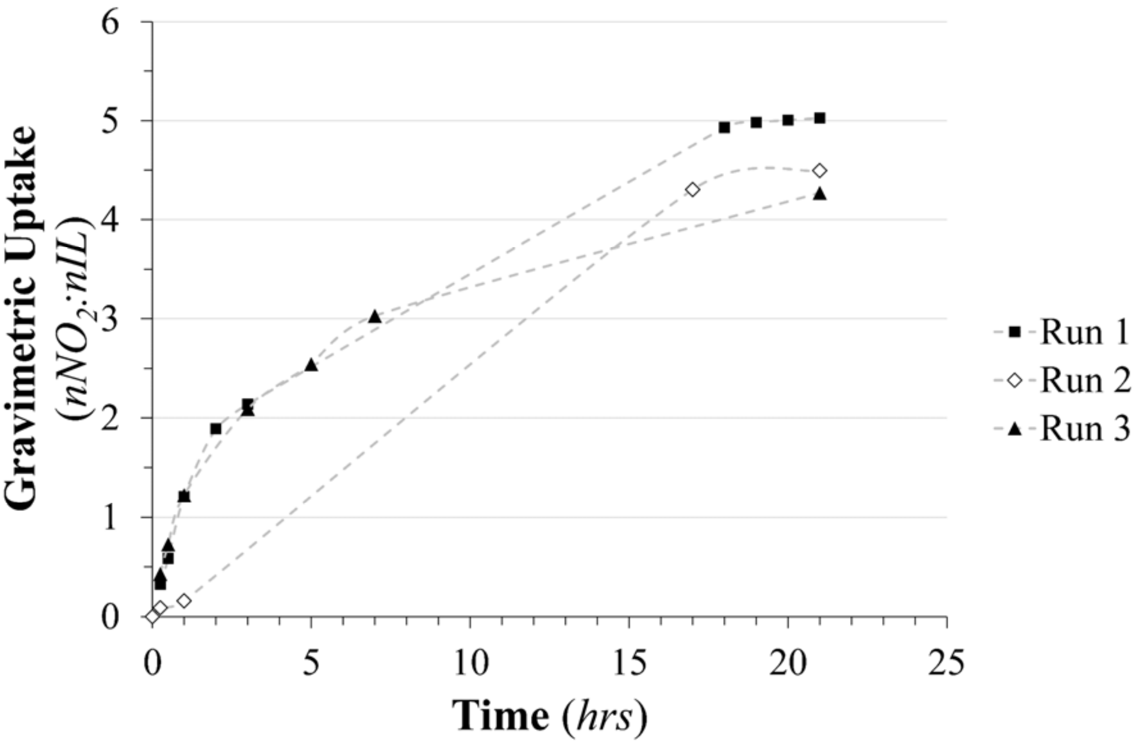


**Figure S1.** Gravimetric uptake of 1% $NO_2$ in argon by [$P_{66614}$][Benzim] over a period of 21 hours.

**Table S1.** Experimental data from the gas absorption rig displaying the $CO_2$ uptake ($nCO_2/nIL$) versus calculated amount of exposure to $NO_2$ ($nNO_2/nIL$), by [$P_{66614}$][Benzim], after 10 cycles of a 2 hour absorption under a feed of 14% $CO_2$ and 0.2% $NO_2$ in argon at 22 °C and atmospheric pressure, and a 2 hour desorption at 90 °C. Weight percent (wt.%) values are displayed in brackets.

| | | **$nCO_2/nIL$ / $nNO_2/nIL$ (*wt.%*)** | | | | | | | | | |
|---|---|---|---|---|---|---|---|---|---|---|---|
| IL | | 1 | 2 | 3 | 4 | 5 | 6 | 7 | 8 | 9 | 10 |
| [$P_{66614}$][Benzim] | $CO_2$ Uptake | 0.79 | 0.78 | 0.66 | 0.69 | 0.52 | 0.49 | 0.45 | 0.40 | 0.31 | 0.31 |
| | | (5.79) | (5.72) | (4.84) | (5.06) | (3.81) | (3.59) | (3.30) | (2.93) | (2.27) | (2.27) |
| | $NO_2$ Exposure | 0.13 | 0.25 | 0.38 | 0.50 | 0.63 | 0.75 | 0.88 | 1.00 | 1.13 | 1.25 |
| | | (1.00) | (1.92) | (2.91) | (3.83) | (4.83) | (5.75) | (6.74) | (7.66) | (8.66) | (9.58) |

u(T) = 0.5 °C; u(nGas:nIL) = 0.04; u(p) = 4 kPa; water content <0.1 wt.%

**[$P_{66614}$][Benzim]:** $^{1}$H NMR (500 MHz, DMSO–$d_6$): δ (ppm) = 0.32-0.40 (s, 12H, $CH_3$(P)), 0.58-0.77 (m, 48H, $CH_2$(P)), 0.95 (s, 8H, $CH_2$(P)), 6.34 (s, 2H, C4 and C5), 6.91 (s, 2H, C3 and C6), 7.36 (s, 1H, C1); $^{13}$C NMR (126 MHz, DMSO–$d_6$): δ (ppm) = 12.96, 16.81, 18.41, 20.19, 21.39, 21.77, 24.96, 26.47, 28.48, 28.81, 30.02, 31.02, 54.77, 114.91, 117.26, 143.02, 147.58.

**[$P_{66614}$][Benzim]** (*$NO_2$ only*)**:** $^{1}$H NMR (500 MHz, DMSO–$d_6$): δ (ppm) = 0.20-0.29 (s, 12H, $CH_3$(P)), 0.57-0.84 (m, 48H, $CH_2$(P)), 1.61 (s, 8H, $CH_2$(P)), 6.57 (s, 2H, C4 and C5), 6.71, 7.10 (s, 2H, C3 and C6), 7.31, 7.65 (s, 1H, C1), 8.23, 8.74, 10.43, 13.19; $^{13}$C NMR (126 MHz, DMSO–$d_6$): δ (ppm) = 12.82, 17.01, 20.25, 21.27, 21.70, 27.81, 28.71, 29.88, 30.95, 114.56, 120.6, 137.62, 140.64, 143.20, 143.71.

**[$P_{66614}$][Benzim]** (*$CO_2$/$NO_2$ co-feed*)**:** $^{1}$H NMR (500 MHz, DMSO–$d_6$): δ (ppm) = 0.22-0.33 (s, 12H, $CH_3$(P)), 0.58-0.70 (m, 48H, $CH_2$(P)), 1.49 (s, 8H, $CH_2$(P)), 6.81 (s, 2H, C4 and C5), 7.07 (s, 2H, C3 and C6), 7.57 (s, 1H, C1), 7.96, 8.45, 10.61, 15.13; $^{13}$C NMR (126 MHz, DMSO–$d_6$): δ (ppm) = 12.89, 16.96, 20.27, 21.32, 21.74, 28.45, 28.74. 29.33, 29.94, 30.99, 114.70, 119.47, 131.71, 139.51, 142.86.

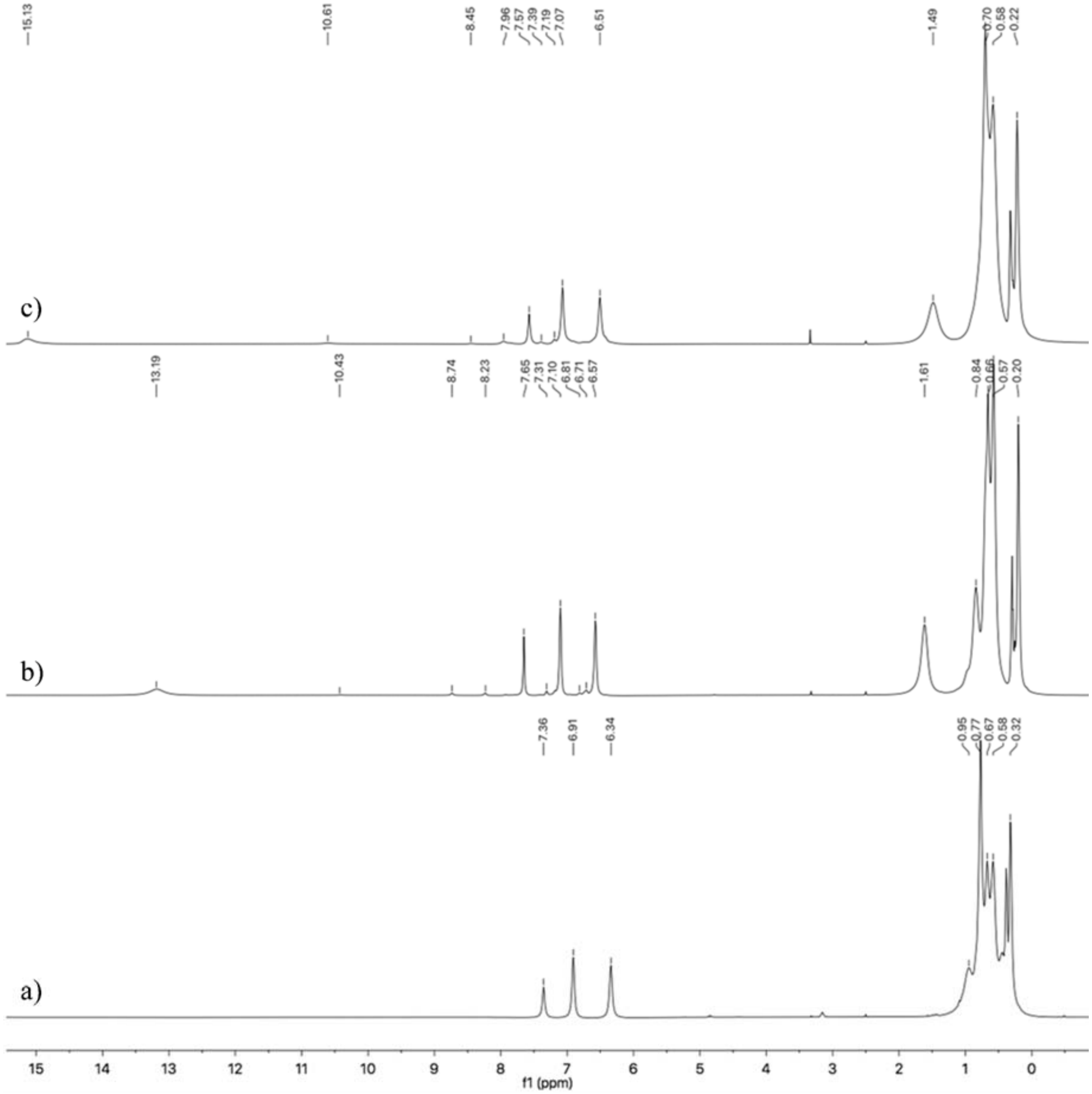


**Figure S2.** $^{1}H$ NMR of [$P_{66614}$][Benzim] under (a) blank IL, (b) 1% $NO_2$ after 6 hrs, (c) 14% $CO_2$ + 0.2% $NO_2$ in Ar after 10 absorption/desorption cycles.

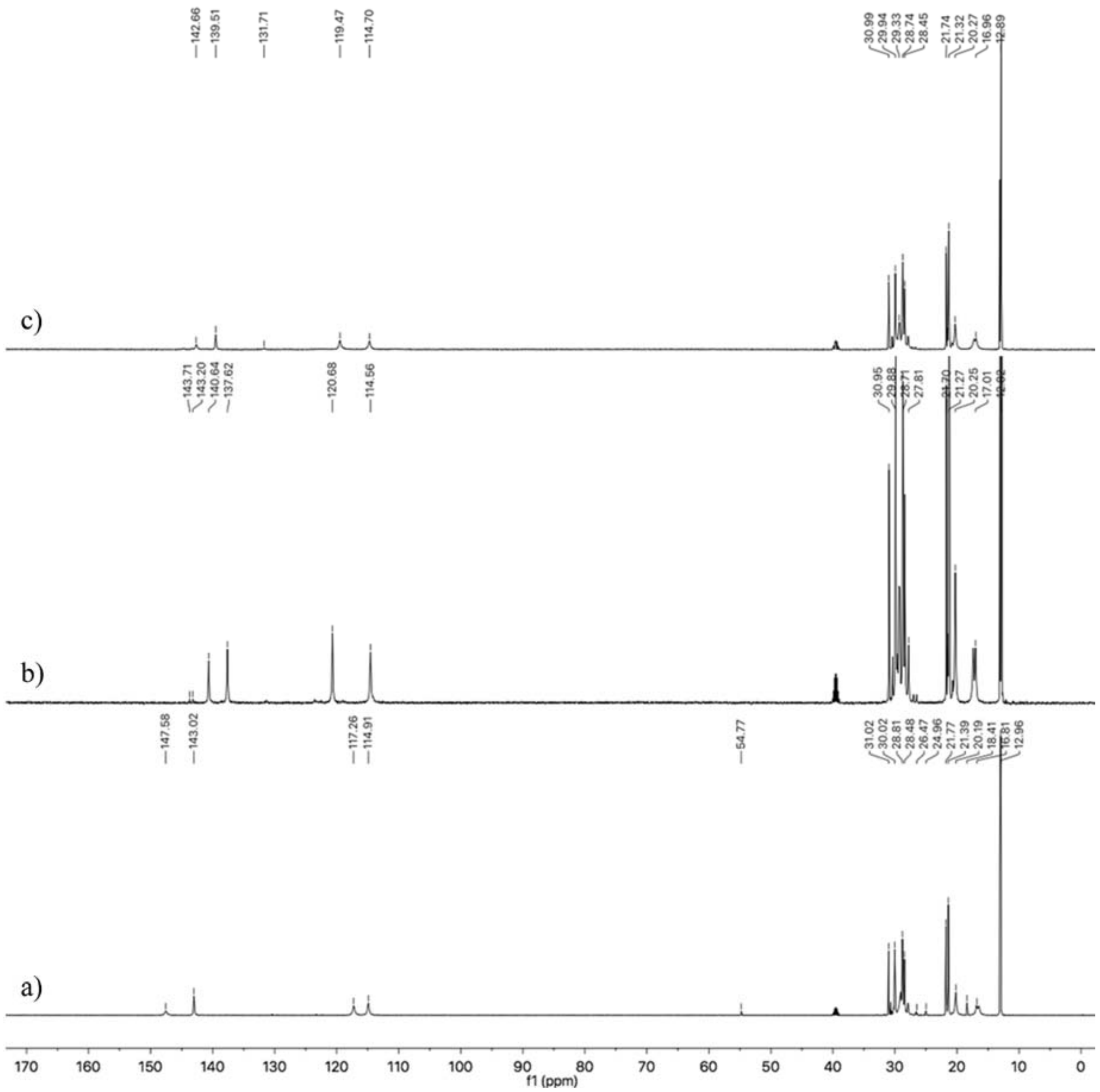


**Figure S3.** $^{13}C$ NMR of [$P_{66614}$][Benzim] under (a) blank IL, (b) 1 % $NO_2$ after 6 hrs, (c) 14% $CO_2$ + 0.2% $NO_2$ in Ar after 10 absorption/desorption cycles.

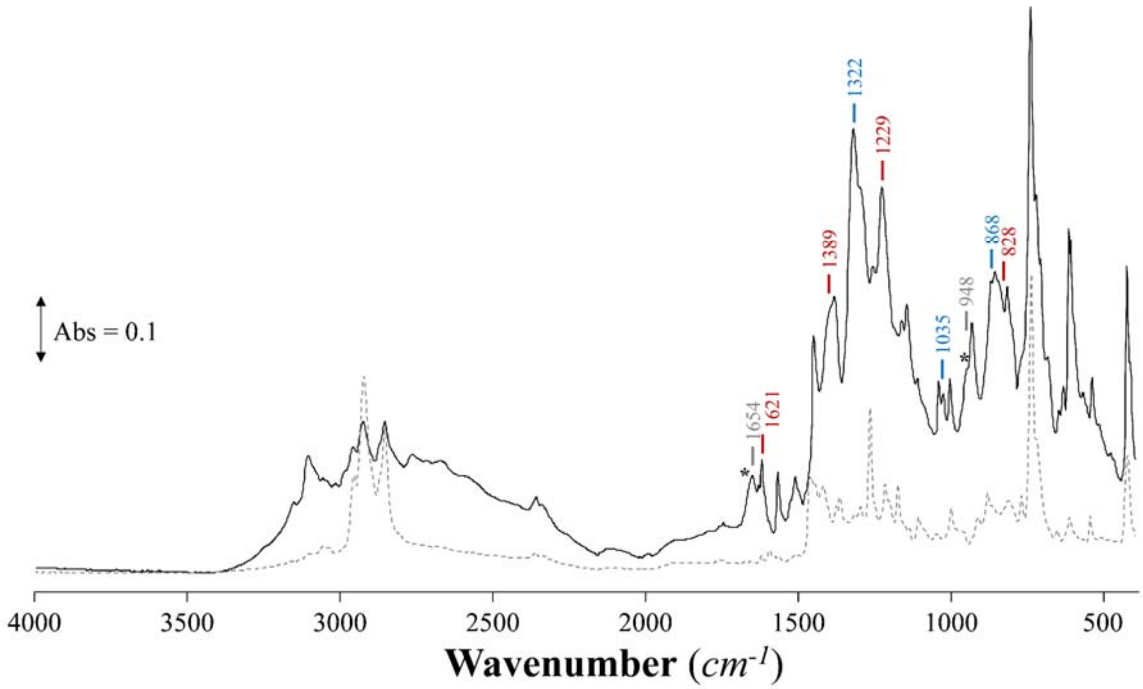


**Figure S4.** ATR-IR spectra of [$P_{66614}$][Benzim] after exposure to 1% $NO_2$ in Ar for 24 hours. Carried out at 22 °C with a flow rate of 40 $cm^3 \cdot min^{-1}$. --- [$P_{66614}$][Benzim] before exposure to the feed. *Small peaks at 1654 and 948 $cm^{-1}$ indicate $HNO_3$ production. *In-silico* modelling found that nitric acid would be expected to protonate the [Benzim]$^-$ anion, leading to further changes in aromaticity of the ring structure.

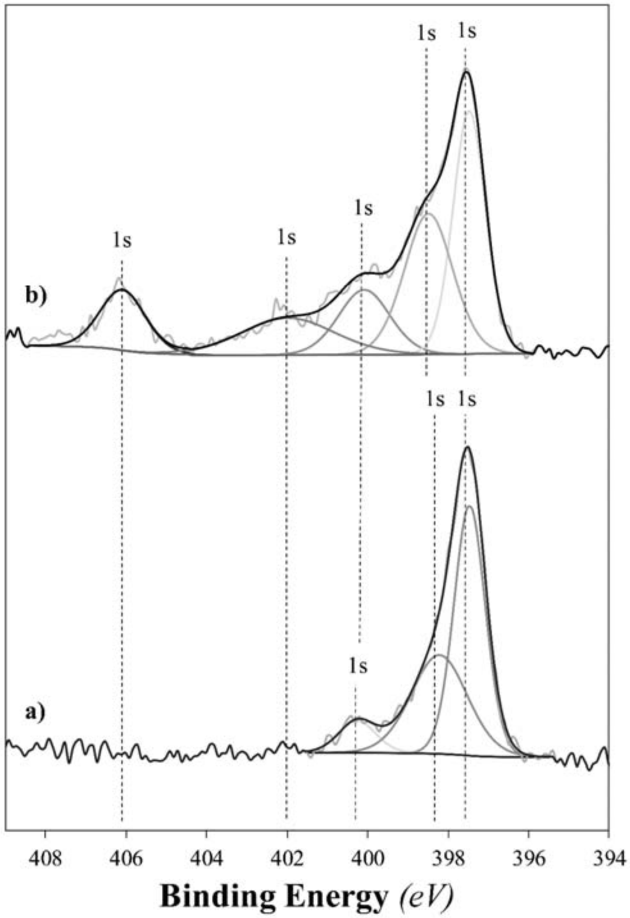

**Figure S5.** XPS spectra of the nitrogen 1s region of [$P_{66614}$][Benzim] (a), and after 10 cycles of 14% $CO_2$ and 0.2% $NO_2$ in argon, with desorption occurring at 90 °C under argon (b). Peaks for the benzimidazolide anion were assigned previously.[1] The spectra were analyzed using CasaXPS and corrected for charging using the C 1s feature at 284.8 eV.

**Table S2.** Gas phase absorption energies for various absorbates to a model of $[P_{3333}]$[Benzim] IL. All energies were obtained at B3LYP/6-311+G* level of theory and are given in $kJ \cdot mol^{-1}$. Enthalpies (**E**) and free energies (**G**) are both given, with the subscript, ZPE, representing the zero-point corrected values. (Physisorption is indicated by …).

| | **E** | **$E_{ZPE}$** | **ΔG** | **Reference** |
|---|---|---|---|---|
| **$[Benzim-CO_2]^-$** | -63.81 | -52.12 | -9.60 | |
| **$[Benzim]^-$…NO** | -32.44 | -29.61 | +5.47 | 1 |
| **$[BenzimNONO]^-$** | -105.95 | -91.09 | -33.15 | 1 |
| **$[Benzim]^-…N_2O_4$** | -128.08 | -107.82 | -7.39 | |
| **$[Benzim]^-…(N_2O_4)_2$** | -249.57 | -210.53 | -28.78 | |
| **$[Benzim-CO_2]^-…(N_2O_4)$** | -175.05 | -147.32 | -5.87 | |

**Table S3.** Solvent phase absorption energies for various absorbates to a model of $[P_{3333}]$[Benzim] IL. All energies were obtained at B3LYP/6-311+G* level of theory and are given in kJ·mol$^{-1}$. Enthalpies (**E**), free energies (**G**) and zero-point energies (ZPE) are provided. (Physisorption is indicated by …).

| | **E** | **$E_{ZPE}$** | **ΔG** | **Reference** |
|---|---|---|---|---|
| **$[Benzim-CO_2]^-$** | -61.53 | -52.58 | -7.56 | |
| **$[Benzim]^-$…NO** | -21.92 | -19.09 | +15.99 | [1] |
| **$[BenzimNONO]^-$** | -87.26 | -72.40 | -14.46 | [1] |
| **$[Benzim]^-$…$N_2O_4$** | -105.95 | -85.69 | +14.74 | |
| **$[Benzim]^-$…$(N_2O_4)_2$** | -213.56 | -174.52 | +22.31 | |
| **$[Benzim-CO_2]^-$…$N_2O_4$** | -158.89 | -131.15 | +10.30 | |

**Table S4.** Grouped Mulliken spin population for absorbates to a model of [$P_{3333}$][Benzim] IL. Total spin populations are grouped into contributions for the cation ([$P_{3333}$]$^+$), anion ([Benzim]$^-$), and various absorbates. (Physisorption is indicated by …).

| | **[$P_{3333}$]** | **[Benzim]** | **Absorbate** |
|---|---|---|---|
| **[Benzim-$CO_2$]$^-$** | 0.00 | 0.00 | 0.00 |
| **[Benzim]$^-$…NO** | 0.01 | -0.02 | 1.00 |
| **[BenzimNONO]$^-$** | 0.00 | 0.00 | 0.00 |
| **[Benzim]$^-$…$N_2O_4$** | 0.00 | 0.00 | 0.00 |

**Table S5.** Grouped Mulliken charges for absorbates to a model of [$P_{3333}$][Benzim] IL. Total Mulliken charges are grouped into contributions for the cation ([$P_{3333}$]$^+$), anion ([Benzim]$^-$), and various absorbates. (Physisorption is indicated by …).

| | **[$P_{3333}$]** | **[Benzim]** | **Absorbate** |
|---|---|---|---|
| **[Benzim-$CO_2$]$^-$** | 1.03 | -0.09 | -0.94 |
| **[Benzim]$^-$…NO** | 1.02 | -0.95 | -0.07 |
| **[BenzimNONO]$^-$** | 0.88 | -0.12 | -0.76 |
| **[Benzim]$^-$…$N_2O_4$** | 0.96 | -1.05 | 0.09 |

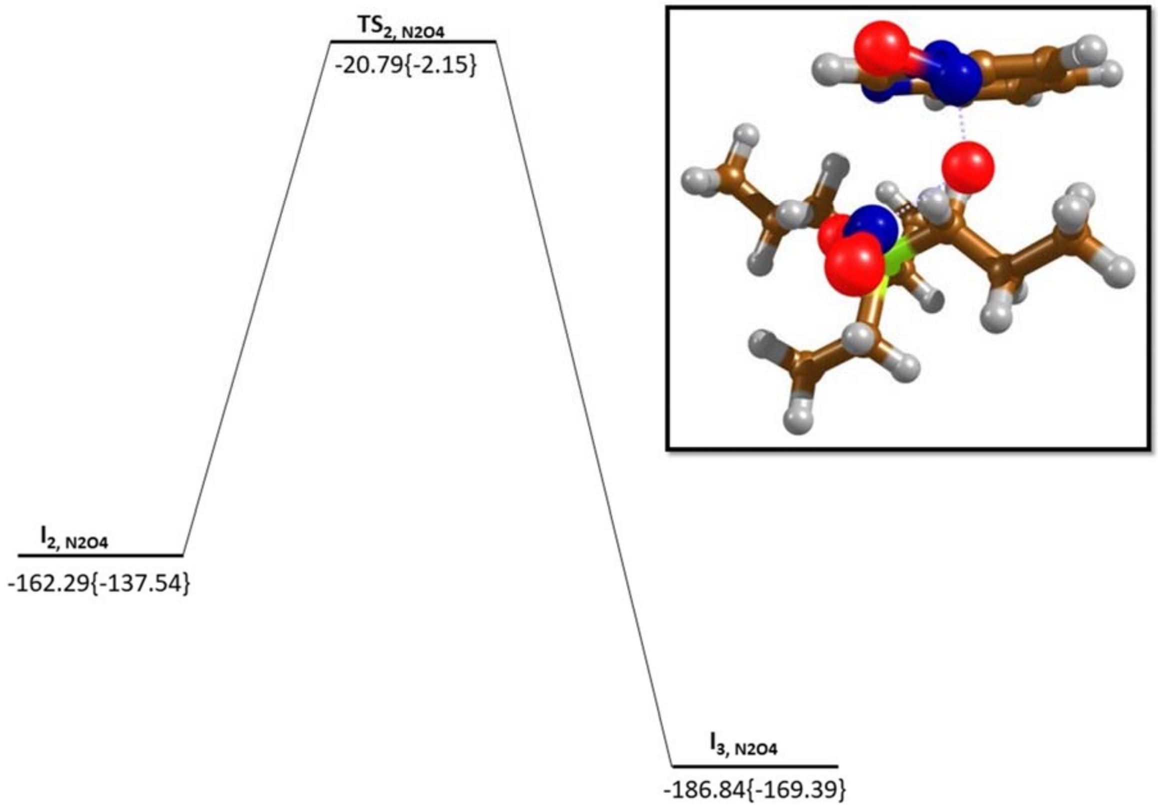


**Figure S6.** Calculated reaction landscapes showing intermediates (I) and transition states (TS) for oxygen transfer from the $NO_2^+$ bound to [$P_{3333}$][Benzim] to free $[NO_2]^-$. Values are given in $kJ \cdot mol^{-1}$ with zero-point corrected gas phase and {solvent} corrected energies calculated at B3LYP/6-311+G* level of theory.

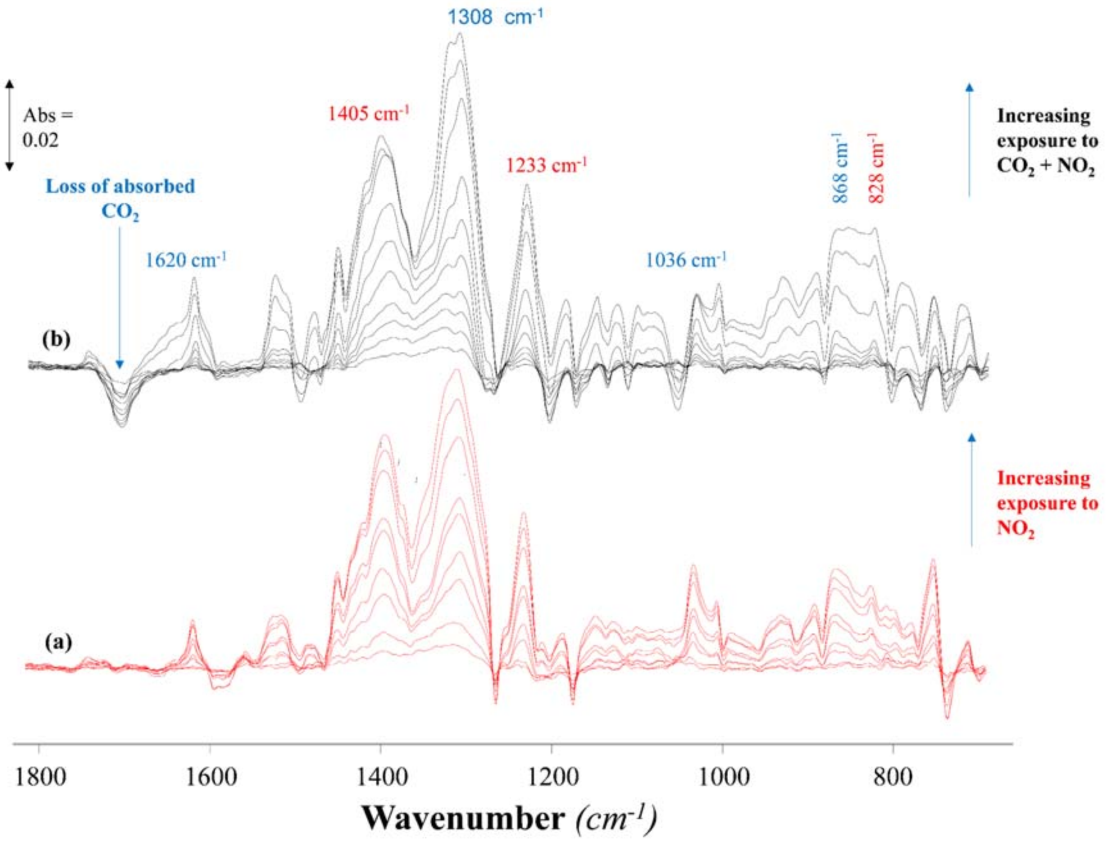


**Figure S7.** ATR-IR subtracted spectra of [$P_{66614}$][Benzim] showing increasing exposure to a feed of (a) 0.2% $NO_2$ in Ar, and (b) 0.2% $NO_2$ + 14% $CO_2$ in Ar. Carried out at 22 °C with a flow rate of 15 $cm^3 \cdot min^{-1}$.

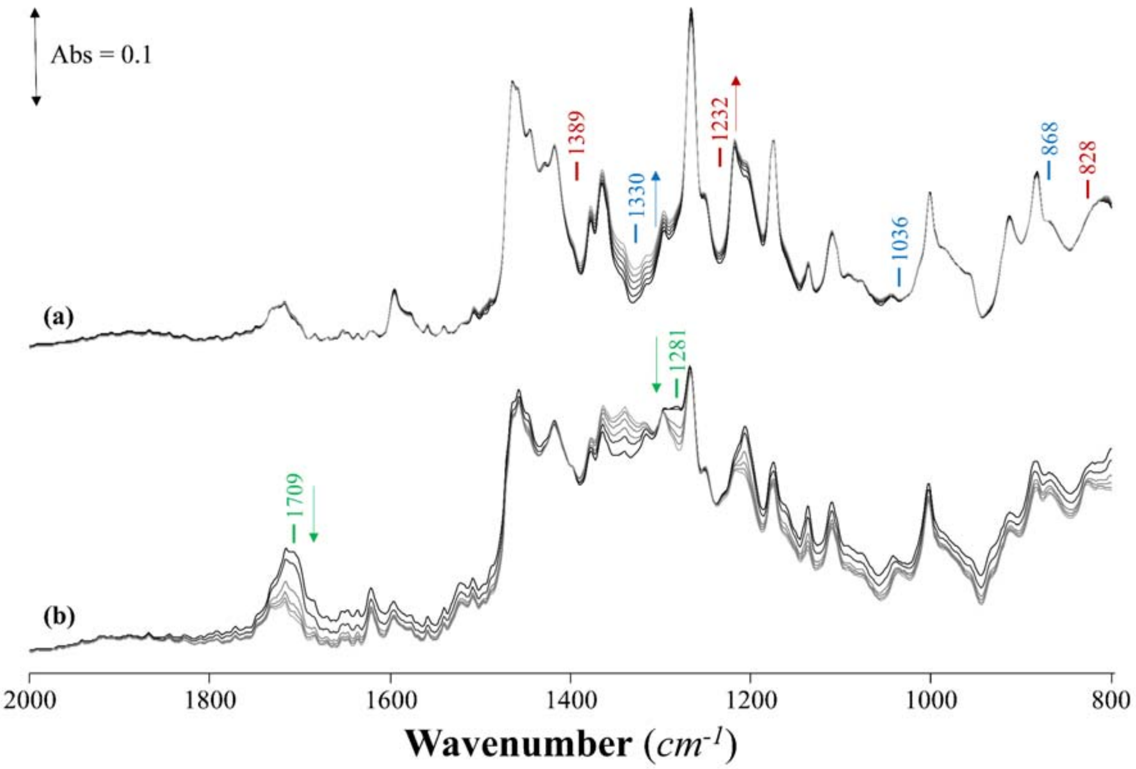


**Figure S8.** ATR-IR subtracted spectra of [$P_{66614}$][Benzim] showing desorption under Ar for 30 mins after exposure to (a) 0.2% $NO_2$ in Ar and (b) 14% $CO_2$ + 0.2% $NO_2$, for 15 mins. Carried out at 90 °C with a flow rate of 40 $cm^3 \cdot min^{-1}$. – $CO_2$ – [Benzim-$NO_2$] – [$P_{66614}$][$NO_2$].

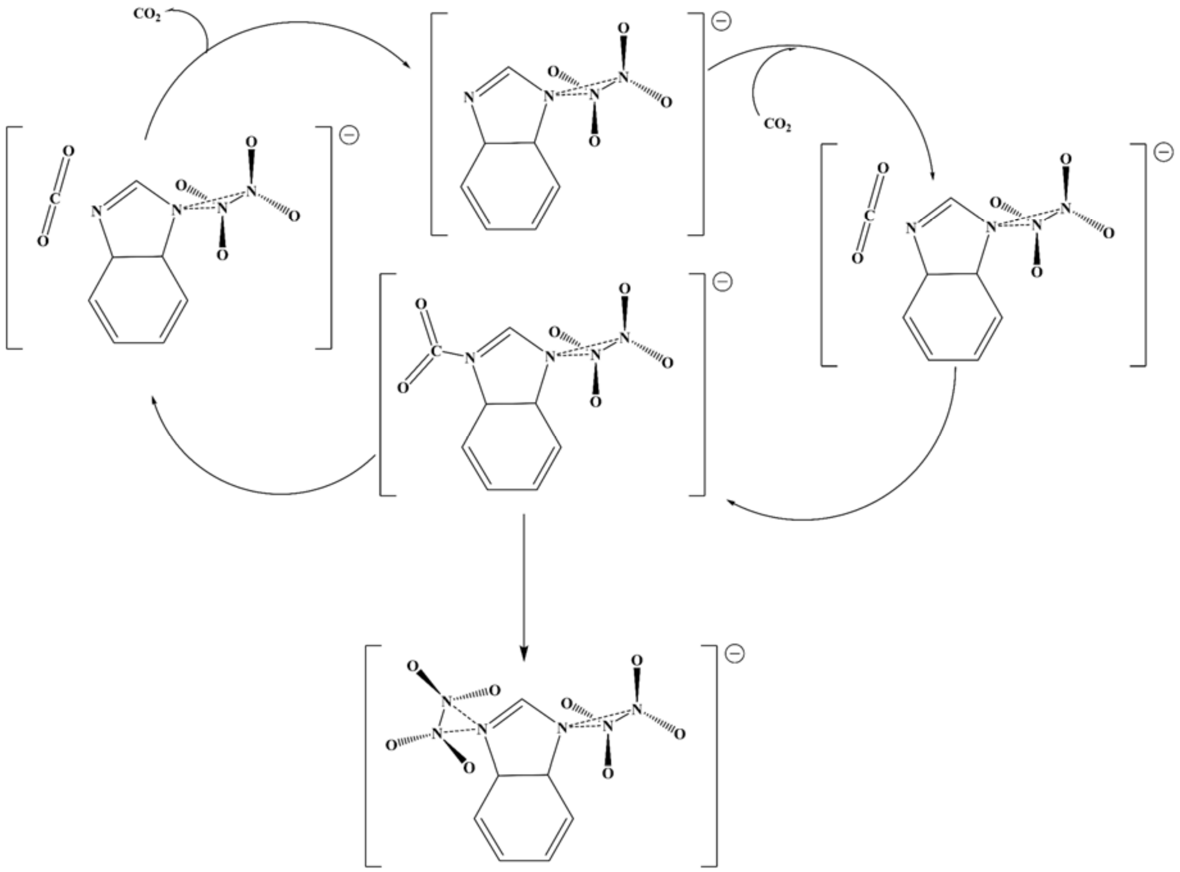


**Figure S9.** Proposed mechanism showing the effect of $N_2O_4$ absorption on the $CO_2$ recyclability of [$P_{66614}$][Benzim].

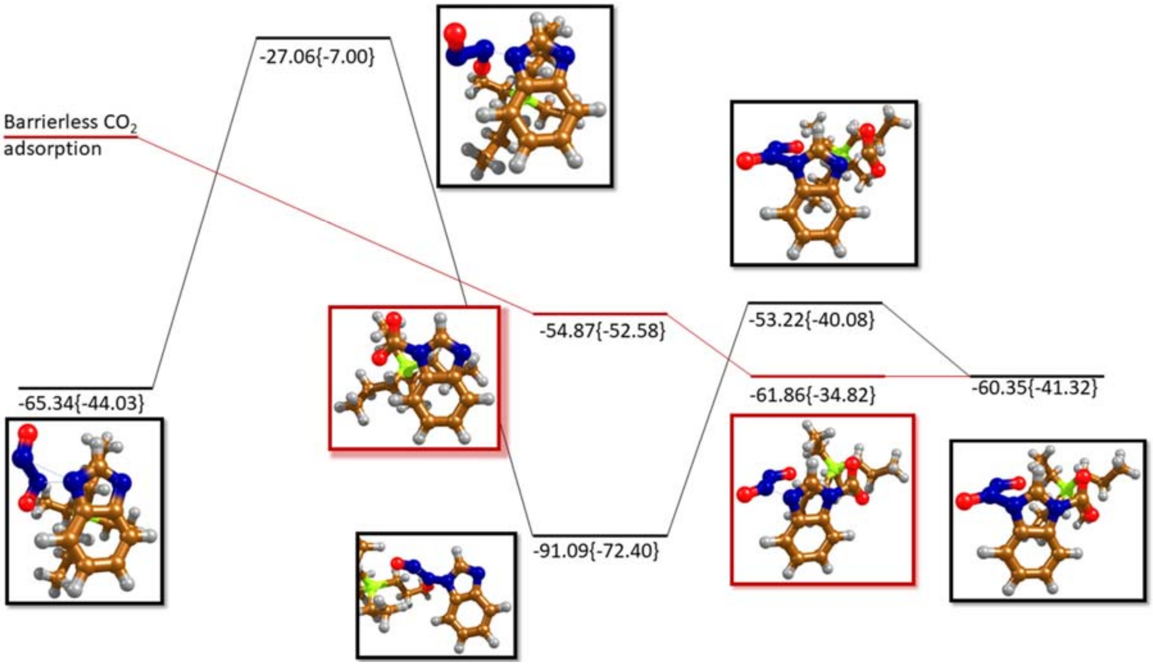


**Figure S10.** Potential energy landscapes of the co-adsorption of $CO_2$/NONO by a truncated [$P_{3333}$][Benzim] model.

## Cartesian Co-Ordinates

B3LYP

$[P_{3333}][Benzim]$

| | X | Y | Z |
|---|---|---|---|
| 15 | -0.159066000 | -1.768462000 | 2.245933000 |
| 6 | 3.062669000 | 0.878148000 | 2.406248000 |
| 1 | 2.797105000 | 1.409396000 | 3.324542000 |
| 1 | 3.853997000 | 0.166734000 | 2.653049000 |
| 6 | 1.849672000 | 0.157019000 | 1.815332000 |
| 1 | 1.077464000 | 0.890061000 | 1.553893000 |
| 1 | 2.143506000 | -0.329848000 | 0.879925000 |
| 1 | 3.468760000 | 1.608449000 | 1.702035000 |
| 6 | 1.318475000 | -0.892512000 | 2.798873000 |
| 1 | 2.064281000 | -1.678371000 | 2.991620000 |
| 1 | 1.086875000 | -0.453858000 | 3.772901000 |
| 6 | -0.670449000 | -3.097737000 | 3.372388000 |
| 1 | -1.431479000 | -3.660870000 | 2.816849000 |
| 1 | 0.187316000 | -3.763589000 | 3.499386000 |
| 6 | -1.215943000 | -2.670745000 | 4.741810000 |
| 1 | -1.996646000 | -1.911723000 | 4.626253000 |
| 1 | -0.411980000 | -2.231777000 | 5.336077000 |
| 6 | -1.799447000 | -3.879336000 | 5.473830000 |
| 1 | -1.059437000 | -4.676050000 | 5.552240000 |
| 1 | -2.676912000 | -4.272654000 | 4.949205000 |
| 1 | -2.104170000 | -3.604988000 | 6.485762000 |
| 6 | 0.161066000 | -2.512784000 | 0.609598000 |
| 1 | -0.793788000 | -2.902259000 | 0.241681000 |
| 1 | 0.441704000 | -1.693203000 | -0.058074000 |
| 6 | 1.235937000 | -3.605186000 | 0.630511000 |
| 1 | 0.904471000 | -4.478997000 | 1.191480000 |
| 1 | 2.161283000 | -3.261015000 | 1.094096000 |
| 1 | 1.455354000 | -3.923250000 | -0.390747000 |
| 6 | -1.536979000 | -0.580825000 | 2.007105000 |
| 1 | -1.413981000 | -0.151996000 | 1.008165000 |
| 1 | -2.460185000 | -1.167733000 | 1.980506000 |
| 6 | -1.624578000 | 0.535140000 | 3.054727000 |
| 1 | -0.734798000 | 1.166039000 | 3.043617000 |
| 1 | -1.741867000 | 0.145125000 | 4.065353000 |
| 1 | -2.484643000 | 1.173260000 | 2.842091000 |
| 1 | 2.645698000 | -5.288430000 | 2.888311000 |
| 6 | 2.438536000 | -4.692583000 | 3.771281000 |
| 7 | 1.789382000 | -5.213824000 | 4.826241000 |
| 6 | 1.759866000 | -4.161293000 | 5.715402000 |
| 6 | 2.404129000 | -3.033732000 | 5.112135000 |
| 6 | 2.493907000 | -1.814626000 | 5.801073000 |
| 6 | 1.928339000 | -1.728964000 | 7.069311000 |
| 1 | 3.015791000 | -0.964909000 | 5.369026000 |
| 1 | 1.994715000 | -0.798170000 | 7.625205000 |
| 6 | 1.296605000 | -2.842447000 | 7.664507000 |
| 1 | 0.883075000 | -2.744079000 | 8.663786000 |
| 6 | 1.216956000 | -4.062711000 | 7.003922000 |
| 1 | 0.745778000 | -4.922442000 | 7.470705000 |
| 7 | 2.827291000 | -3.397267000 | 3.849788000 |

$[P_{3333}][Benzim]CO_2$

| | X | Y | Z |
|---|---|---|---|
| 15 | -0.152713000 | -1.683088000 | 2.150301000 |
| 6 | 3.028318000 | 0.982835000 | 2.540408000 |
| 1 | 2.791023000 | 1.418240000 | 3.515663000 |
| 1 | 3.816663000 | 0.240983000 | 2.683528000 |
| 6 | 1.796110000 | 0.333379000 | 1.908844000 |
| 1 | 1.022838000 | 1.089185000 | 1.727758000 |
| 1 | 2.072826000 | -0.074262000 | 0.932197000 |
| 1 | 3.420570000 | 1.780391000 | 1.904805000 |
| 6 | 1.275948000 | -0.798309000 | 2.804396000 |
| 1 | 2.067710000 | -1.548883000 | 2.932305000 |
| 1 | 1.010912000 | -0.438756000 | 3.802617000 |
| 6 | -0.562386000 | -3.135354000 | 3.168699000 |
| 1 | -1.260724000 | -3.729917000 | 2.567924000 |
| 1 | 0.350906000 | -3.726932000 | 3.268748000 |
| 6 | -1.163572000 | -2.836103000 | 4.550604000 |
| 1 | -1.981408000 | -2.114887000 | 4.458203000 |

| | | | |
|---|---|---|---|
| 1 | -0.402621000 | -2.384253000 | 5.191035000 |
| 6 | -1.702996000 | -4.109224000 | 5.203835000 |
| 1 | -0.926038000 | -4.867291000 | 5.308212000 |
| 1 | -2.522550000 | -4.534227000 | 4.615160000 |
| 1 | -2.087682000 | -3.889753000 | 6.202254000 |
| 6 | 0.165337000 | -2.243482000 | 0.444375000 |
| 1 | -0.775456000 | -2.674686000 | 0.084386000 |
| 1 | 0.338760000 | -1.340529000 | -0.148893000 |
| 6 | 1.328101000 | -3.230819000 | 0.303581000 |
| 1 | 1.151280000 | -4.161237000 | 0.842987000 |
| 1 | 2.267768000 | -2.824409000 | 0.677183000 |
| 1 | 1.460843000 | -3.484876000 | -0.749779000 |
| 6 | -1.604484000 | -0.563595000 | 2.057772000 |
| 1 | -1.551546000 | -0.066422000 | 1.084968000 |
| 1 | -2.495464000 | -1.198312000 | 2.030552000 |
| 6 | -1.701936000 | 0.478133000 | 3.178132000 |
| 1 | -0.857984000 | 1.168990000 | 3.156314000 |
| 1 | -1.733885000 | 0.022758000 | 4.167970000 |
| 1 | -2.613069000 | 1.066883000 | 3.054346000 |
| 1 | 2.198103000 | -6.343858000 | 3.830025000 |
| 6 | 2.103086000 | -5.519162000 | 4.519424000 |
| 7 | 1.460689000 | -5.541157000 | 5.663967000 |
| 6 | 1.615708000 | -4.258773000 | 6.178604000 |
| 6 | 2.381215000 | -3.465892000 | 5.285438000 |
| 6 | 2.694189000 | -2.135958000 | 5.573293000 |
| 6 | 2.183896000 | -1.603751000 | 6.753533000 |
| 1 | 3.321208000 | -1.560179000 | 4.907982000 |
| 1 | 2.413317000 | -0.574666000 | 7.011924000 |
| 6 | 1.399881000 | -2.374232000 | 7.633864000 |
| 1 | 1.028652000 | -1.921925000 | 8.547783000 |
| 6 | 1.117604000 | -3.707563000 | 7.362455000 |
| 1 | 0.531074000 | -4.315181000 | 8.042866000 |
| 7 | 2.675634000 | -4.305747000 | 4.216404000 |
| 6 | 3.313867000 | -3.978261000 | 2.886591000 |
| 8 | 3.428130000 | -4.946651000 | 2.143030000 |
| 8 | 3.560803000 | -2.765237000 | 2.749161000 |

## $[P_{3333}]$[Benzim]$2CO_2$

| | X | Y | Z |
|---|---|---|---|
| 15 | -1.056374000 | -2.127459000 | 1.787895000 |
| 6 | 2.934618000 | -1.213949000 | 0.936764000 |
| 1 | 3.308887000 | -1.132976000 | 1.959846000 |
| 1 | 3.266810000 | -2.176333000 | 0.541419000 |
| 6 | 1.410258000 | -1.092003000 | 0.891118000 |
| 1 | 1.108849000 | -0.102605000 | 1.249664000 |
| 1 | 1.066984000 | -1.164755000 | -0.146139000 |
| 1 | 3.404604000 | -0.427024000 | 0.342328000 |
| 6 | 0.752112000 | -2.189370000 | 1.738132000 |
| 1 | 1.013435000 | -3.173462000 | 1.347847000 |
| 1 | 1.098077000 | -2.161209000 | 2.775146000 |
| 6 | -1.745952000 | -3.501064000 | 2.752017000 |
| 1 | -2.818567000 | -3.523132000 | 2.525530000 |
| 1 | -1.311417000 | -4.412231000 | 2.325593000 |
| 6 | -1.507087000 | -3.447788000 | 4.269286000 |
| 1 | -2.064239000 | -2.612916000 | 4.706098000 |
| 1 | -0.452306000 | -3.268788000 | 4.489648000 |
| 6 | -1.951322000 | -4.755691000 | 4.925948000 |
| 1 | -1.409554000 | -5.608720000 | 4.509539000 |
| 1 | -3.019467000 | -4.939818000 | 4.774720000 |
| 1 | -1.764419000 | -4.728616000 | 6.001666000 |
| 6 | -1.704491000 | -2.257019000 | 0.087080000 |
| 1 | -2.796261000 | -2.253695000 | 0.165901000 |
| 1 | -1.426087000 | -1.339050000 | -0.438562000 |
| 6 | -1.205540000 | -3.499483000 | -0.664052000 |
| 1 | -1.416877000 | -4.427355000 | -0.132160000 |
| 1 | -0.128296000 | -3.465054000 | -0.833763000 |
| 1 | -1.688273000 | -3.551761000 | -1.641537000 |
| 6 | -1.579806000 | -0.518860000 | 2.487230000 |
| 1 | -1.540367000 | 0.214533000 | 1.676612000 |
| 1 | -2.634464000 | -0.625043000 | 2.759771000 |
| 6 | -0.743499000 | -0.045528000 | 3.684561000 |
| 1 | 0.288910000 | 0.159422000 | 3.400249000 |
| 1 | -0.716145000 | -0.775947000 | 4.491314000 |

| | | | |
|---|---|---|---|
| 1 | -1.166849000 | 0.879532000 | 4.080708000 |
| 6 | 4.093247000 | -5.442074000 | 0.233436000 |
| 6 | 3.388199000 | -5.136348000 | 1.398229000 |
| 6 | 3.956873000 | -4.343295000 | 2.418721000 |
| 6 | 5.251835000 | -3.835081000 | 2.312076000 |
| 6 | 5.949954000 | -4.144314000 | 1.152164000 |
| 6 | 5.381268000 | -4.931204000 | 0.132342000 |
| 7 | 2.088840000 | -5.448789000 | 1.815024000 |
| 6 | 1.903200000 | -4.863827000 | 3.002683000 |
| 7 | 2.977623000 | -4.190346000 | 3.404622000 |
| 1 | 3.638907000 | -6.043324000 | -0.540909000 |
| 1 | 5.668879000 | -3.227693000 | 3.102807000 |
| 1 | 6.960557000 | -3.770287000 | 1.028722000 |
| 1 | 5.965400000 | -5.146496000 | -0.755889000 |
| 1 | 0.974857000 | -4.894836000 | 3.542094000 |
| 8 | -0.172801000 | -5.922579000 | 1.568153000 |
| 6 | 0.885761000 | -6.004686000 | 0.943726000 |
| 8 | 1.238347000 | -6.342919000 | -0.168367000 |
| 8 | 1.738634000 | -2.867551000 | 4.887917000 |
| 6 | 2.906938000 | -3.041286000 | 4.556714000 |
| 8 | 4.000229000 | -2.579766000 | 4.790683000 |

## [P3333][Benzim]$N_2O_4$

| | X | Y | Z |
|---|---|---|---|
| 15 | -0.106862000 | -1.827890000 | 2.279366000 |
| 6 | 2.988138000 | 0.969451000 | 2.267279000 |
| 1 | 2.669085000 | 1.576875000 | 3.119143000 |
| 1 | 3.802306000 | 0.324909000 | 2.606359000 |
| 6 | 1.828973000 | 0.135658000 | 1.719116000 |
| 1 | 1.028415000 | 0.798804000 | 1.370782000 |
| 1 | 2.171637000 | -0.422907000 | 0.842374000 |
| 1 | 3.383209000 | 1.644790000 | 1.504442000 |
| 6 | 1.322362000 | -0.845166000 | 2.783302000 |
| 1 | 2.105326000 | -1.568105000 | 3.058971000 |
| 1 | 1.045784000 | -0.331301000 | 3.708247000 |
| 6 | -0.554685000 | -3.105392000 | 3.489733000 |
| 1 | -1.269020000 | -3.753177000 | 2.968351000 |
| 1 | 0.337451000 | -3.708399000 | 3.671547000 |
| 6 | -1.146619000 | -2.597462000 | 4.812547000 |
| 1 | -1.904738000 | -1.830950000 | 4.623376000 |
| 1 | -0.357390000 | -2.137626000 | 5.410673000 |
| 6 | -1.792632000 | -3.741509000 | 5.592639000 |
| 1 | -1.081209000 | -4.546378000 | 5.769430000 |
| 1 | -2.649261000 | -4.156012000 | 5.051250000 |
| 1 | -2.142727000 | -3.390709000 | 6.565876000 |
| 6 | 0.227605000 | -2.648734000 | 0.683428000 |
| 1 | -0.709866000 | -3.124799000 | 0.377559000 |
| 1 | 0.425065000 | -1.854012000 | -0.042002000 |
| 6 | 1.372959000 | -3.664319000 | 0.723092000 |
| 1 | 1.145743000 | -4.502849000 | 1.380284000 |
| 1 | 2.302162000 | -3.212709000 | 1.072280000 |
| 1 | 1.541581000 | -4.060995000 | -0.280236000 |
| 6 | -1.547358000 | -0.728524000 | 1.980487000 |
| 1 | -1.480199000 | -0.397000000 | 0.940382000 |
| 1 | -2.440725000 | -1.356964000 | 2.046465000 |
| 6 | -1.657639000 | 0.482246000 | 2.914611000 |
| 1 | -0.807606000 | 1.156355000 | 2.801040000 |
| 1 | -1.714412000 | 0.193092000 | 3.963912000 |
| 1 | -2.560610000 | 1.048645000 | 2.677619000 |
| 1 | 3.191563000 | -4.905312000 | 2.824741000 |
| 6 | 2.857251000 | -4.422920000 | 3.735735000 |
| 7 | 2.200177000 | -5.114319000 | 4.686246000 |
| 6 | 1.974986000 | -4.169771000 | 5.667342000 |
| 6 | 2.518276000 | -2.925102000 | 5.218504000 |
| 6 | 2.408201000 | -1.774538000 | 6.011036000 |
| 6 | 1.759274000 | -1.880527000 | 7.237708000 |
| 1 | 2.842374000 | -0.831910000 | 5.689208000 |
| 1 | 1.671993000 | -1.006324000 | 7.875994000 |
| 6 | 1.235239000 | -3.112004000 | 7.683507000 |
| 1 | 0.750567000 | -3.159887000 | 8.653802000 |
| 6 | 1.341470000 | -4.264376000 | 6.912486000 |
| 1 | 0.949090000 | -5.213177000 | 7.264310000 |
| 7 | 3.077247000 | -3.114550000 | 3.968186000 |
| 7 | 0.246925000 | -6.815974000 | 3.927357000 |
| 8 | -0.365227000 | -6.912187000 | 4.942481000 |
| 8 | -0.020697000 | -6.334116000 | 2.864836000 |

| | | | |
|---|---|---|---|
| 7 | 1.815519000 | -7.629326000 | 3.965191000 |
| 8 | 2.397664000 | -7.541485000 | 2.933087000 |
| 8 | 2.001920000 | -8.205401000 | 4.982294000 |

## [$P_{3333}$][Benzim] $N_2O_4$_TS1

| | X | Y | Z |
|---|---|---|---|
| 15 | -0.530483000 | -1.952574000 | 2.328559000 |
| 6 | 3.104773000 | 0.056402000 | 2.739303000 |
| 1 | 2.856690000 | 0.600709000 | 3.654867000 |
| 1 | 3.786461000 | -0.751966000 | 3.008086000 |
| 6 | 1.842331000 | -0.484475000 | 2.066928000 |
| 1 | 1.195322000 | 0.352150000 | 1.779432000 |
| 1 | 2.113014000 | -0.995897000 | 1.137961000 |
| 1 | 3.627779000 | 0.744924000 | 2.070857000 |
| 6 | 1.086202000 | -1.462982000 | 2.980068000 |
| 1 | 1.655581000 | -2.380876000 | 3.122779000 |
| 1 | 0.935714000 | -1.046942000 | 3.979900000 |
| 6 | -1.349251000 | -3.167867000 | 3.397771000 |
| 1 | -2.177089000 | -3.580964000 | 2.809829000 |
| 1 | -0.630206000 | -3.981696000 | 3.539231000 |
| 6 | -1.851126000 | -2.645034000 | 4.749938000 |
| 1 | -2.600709000 | -1.861269000 | 4.601956000 |
| 1 | -1.020548000 | -2.202180000 | 5.304760000 |
| 6 | -2.461217000 | -3.779497000 | 5.573336000 |
| 1 | -1.740172000 | -4.583581000 | 5.730033000 |
| 1 | -3.332988000 | -4.210413000 | 5.072319000 |
| 1 | -2.784341000 | -3.418091000 | 6.552416000 |
| 6 | -0.318374000 | -2.686695000 | 0.671948000 |
| 1 | -1.318413000 | -2.972045000 | 0.329033000 |
| 1 | 0.021272000 | -1.883707000 | 0.011214000 |
| 6 | 0.642966000 | -3.880087000 | 0.637154000 |
| 1 | 0.331766000 | -4.688140000 | 1.297739000 |
| 1 | 1.654284000 | -3.594047000 | 0.929739000 |
| 1 | 0.695558000 | -4.275431000 | -0.378711000 |
| 6 | -1.580762000 | -0.463476000 | 2.123989000 |
| 1 | -1.308805000 | -0.012755000 | 1.165799000 |
| 1 | -2.610553000 | -0.817988000 | 2.017189000 |
| 6 | -1.467754000 | 0.570762000 | 3.250813000 |
| 1 | -0.461314000 | 0.986246000 | 3.315345000 |
| 1 | -1.718452000 | 0.151107000 | 4.224591000 |
| 1 | -2.153375000 | 1.398519000 | 3.059442000 |
| 1 | 4.258989000 | -4.488200000 | 2.668184000 |
| 6 | 3.778893000 | -4.028445000 | 3.518768000 |
| 7 | 2.666408000 | -4.580967000 | 4.075660000 |
| 6 | 2.287207000 | -3.783108000 | 5.134006000 |
| 6 | 3.249389000 | -2.736411000 | 5.148895000 |
| 6 | 3.137936000 | -1.704642000 | 6.087263000 |
| 6 | 2.076384000 | -1.746283000 | 6.983480000 |
| 1 | 3.869781000 | -0.904369000 | 6.110588000 |
| 1 | 1.974385000 | -0.963614000 | 7.728283000 |
| 6 | 1.144761000 | -2.804823000 | 6.967996000 |
| 1 | 0.349846000 | -2.820376000 | 7.706813000 |
| 6 | 1.235930000 | -3.843665000 | 6.049098000 |
| 1 | 0.550325000 | -4.679378000 | 6.048019000 |
| 7 | 4.169324000 | -2.920237000 | 4.123787000 |
| 7 | 1.805750000 | -5.833303000 | 3.373304000 |
| 8 | 0.590896000 | -5.605683000 | 3.496496000 |
| 8 | 2.385159000 | -6.194911000 | 2.359620000 |
| 7 | 2.245122000 | -7.090685000 | 4.770248000 |
| 8 | 3.247120000 | -7.734874000 | 4.592888000 |
| 8 | 1.470662000 | -7.132442000 | 5.698765000 |

## [$P_{3333}$][Benzim] $NO_2$_$NO_2$

| | X | Y | Z |
|---|---|---|---|
| 15 | -1.625095000 | 1.550530000 | -0.482485000 |
| 6 | 2.186728000 | 3.166029000 | 0.097015000 |
| 1 | 2.226542000 | 3.215945000 | 1.189131000 |
| 1 | 2.759327000 | 2.292936000 | -0.222530000 |
| 6 | 0.746857000 | 3.069382000 | -0.406900000 |
| 1 | 0.193935000 | 3.974866000 | -0.130205000 |
| 1 | 0.758335000 | 3.012277000 | -1.497545000 |
| 1 | 2.680398000 | 4.056583000 | -0.299867000 |
| 6 | 0.054732000 | 1.824427000 | 0.155279000 |
| 1 | 0.615332000 | 0.930043000 | -0.104050000 |
| 1 | -0.012521000 | 1.859511000 | 1.245861000 |
| 6 | -2.290014000 | 0.036675000 | 0.281230000 |
| 1 | -1.730364000 | -0.791261000 | -0.149858000 |
| 1 | -1.979055000 | 0.097848000 | 1.328592000 |

| | | | |
|---|---|---|---|
| 6 | -3.802233000 | -0.193379000 | 0.194319000 |
| 1 | -4.098816000 | -0.342877000 | -0.845782000 |
| 1 | -4.353263000 | 0.682778000 | 0.552445000 |
| 6 | -4.202914000 | -1.417772000 | 1.020224000 |
| 1 | -3.969319000 | -1.273563000 | 2.079182000 |
| 1 | -3.663908000 | -2.308280000 | 0.689608000 |
| 1 | -5.274661000 | -1.615043000 | 0.937026000 |
| 6 | -1.664644000 | 1.653901000 | -2.306419000 |
| 1 | -1.878488000 | 2.700895000 | -2.546895000 |
| 1 | -0.643921000 | 1.421390000 | -2.618986000 |
| 6 | -2.652195000 | 0.713170000 | -2.996820000 |
| 1 | -3.691922000 | 0.913105000 | -2.726067000 |
| 1 | -2.409803000 | -0.323720000 | -2.764276000 |
| 1 | -2.564894000 | 0.837649000 | -4.078322000 |
| 6 | -2.659444000 | 2.945684000 | 0.113813000 |
| 1 | -2.245342000 | 3.855736000 | -0.328972000 |
| 1 | -3.653922000 | 2.819876000 | -0.324228000 |
| 6 | -2.742385000 | 3.075035000 | 1.636276000 |
| 1 | -1.758973000 | 3.241379000 | 2.080389000 |
| 1 | -3.171208000 | 2.183802000 | 2.098891000 |
| 1 | -3.373619000 | 3.922872000 | 1.910758000 |
| 8 | 2.371969000 | -3.605700000 | -1.464368000 |
| 7 | 2.643044000 | -2.698743000 | -0.707905000 |
| 8 | 3.665007000 | -2.049007000 | -0.647826000 |
| 7 | 1.666661000 | -2.399510000 | 0.263786000 |
| 6 | 1.716881000 | -1.405056000 | 1.245270000 |
| 6 | 0.470231000 | -1.533105000 | 1.897712000 |
| 7 | -0.302820000 | -2.536609000 | 1.304415000 |
| 6 | 0.415111000 | -3.007414000 | 0.333960000 |
| 1 | 0.141563000 | -3.775611000 | -0.369571000 |
| 6 | 2.662279000 | -0.457156000 | 1.619147000 |
| 1 | 3.603324000 | -0.362328000 | 1.098556000 |
| 6 | 2.325784000 | 0.368086000 | 2.690865000 |
| 1 | 3.035101000 | 1.122318000 | 3.012592000 |
| 6 | 1.099654000 | 0.245907000 | 3.361605000 |
| 1 | 0.885996000 | 0.900175000 | 4.200200000 |
| 6 | 0.158943000 | -0.704362000 | 2.973319000 |
| 1 | -0.787116000 | -0.815790000 | 3.491439000 |
| 8 | 1.309304000 | 0.493381000 | -2.447242000 |
| 7 | 1.049619000 | -0.651798000 | -2.031794000 |
| 8 | -0.117755000 | -0.810243000 | -1.552445000 |

## [$P_{3333}$][Benzim] $NO_2$_$NO_2$_TS2

| | X | Y | Z |
|---|---|---|---|
| 15 | 0.171746000 | -2.151489000 | 1.680919000 |
| 6 | 4.206777000 | -1.092394000 | 1.982038000 |
| 1 | 4.296457000 | -1.102208000 | 3.069391000 |
| 1 | 4.659921000 | -2.014240000 | 1.612051000 |
| 6 | 2.742752000 | -0.997675000 | 1.550941000 |
| 1 | 2.312585000 | -0.059210000 | 1.920891000 |
| 1 | 2.686483000 | -0.956010000 | 0.457951000 |
| 1 | 4.779772000 | -0.246582000 | 1.592946000 |
| 6 | 1.939839000 | -2.195032000 | 2.074460000 |
| 1 | 2.335723000 | -3.137835000 | 1.693386000 |
| 1 | 2.016943000 | -2.277633000 | 3.160864000 |
| 6 | -0.585073000 | -3.761957000 | 2.023490000 |
| 1 | -0.308554000 | -4.412777000 | 1.187199000 |
| 1 | -0.047731000 | -4.161082000 | 2.900866000 |
| 6 | -2.099166000 | -3.782670000 | 2.271664000 |
| 1 | -2.655632000 | -3.378377000 | 1.420857000 |
| 1 | -2.339963000 | -3.147057000 | 3.128843000 |
| 6 | -2.563711000 | -5.211238000 | 2.562183000 |
| 1 | -2.024385000 | -5.627527000 | 3.415972000 |
| 1 | -2.383334000 | -5.866424000 | 1.705008000 |
| 1 | -3.633321000 | -5.239412000 | 2.784520000 |
| 6 | -0.021911000 | -1.693447000 | -0.079506000 |
| 1 | 0.387673000 | -0.685760000 | -0.189931000 |
| 1 | 0.637988000 | -2.362574000 | -0.638026000 |
| 6 | -1.449175000 | -1.765471000 | -0.627910000 |
| 1 | -2.142140000 | -1.136836000 | -0.064512000 |
| 1 | -1.832615000 | -2.786509000 | -0.618966000 |
| 1 | -1.463497000 | -1.419923000 | -1.663519000 |
| 6 | -0.633108000 | -0.859724000 | 2.688192000 |
| 1 | -0.218078000 | 0.101093000 | 2.370246000 |
| 1 | -1.692189000 | -0.848936000 | 2.417814000 |
| 6 | -0.448483000 | -1.066593000 | 4.195878000 |
| 1 | 0.598641000 | -1.007942000 | 4.492909000 |

| | | | |
|---|---|---|---|
| 1 | -0.818041000 | -2.039553000 | 4.522329000 |
| 1 | -0.997259000 | -0.297579000 | 4.743205000 |
| 8 | 4.166867000 | -6.573498000 | 0.737275000 |
| 7 | 4.685288000 | -5.545779000 | 1.012910000 |
| 8 | 4.362188000 | -4.471878000 | 0.319269000 |
| 7 | 3.458443000 | -5.061851000 | 3.234349000 |
| 6 | 3.518185000 | -4.135024000 | 4.250829000 |
| 6 | 2.213803000 | -4.029317000 | 4.834633000 |
| 7 | 1.365119000 | -4.890242000 | 4.170167000 |
| 6 | 2.176723000 | -5.461834000 | 3.253869000 |
| 1 | 1.807844000 | -6.206730000 | 2.555249000 |
| 6 | 4.575906000 | -3.347135000 | 4.720769000 |
| 1 | 5.567091000 | -3.436494000 | 4.286889000 |
| 6 | 4.319445000 | -2.447690000 | 5.749721000 |
| 1 | 5.123979000 | -1.827086000 | 6.132683000 |
| 6 | 3.033177000 | -2.334687000 | 6.320211000 |
| 1 | 2.875392000 | -1.633845000 | 7.134742000 |
| 6 | 1.975797000 | -3.123863000 | 5.878288000 |
| 1 | 0.994720000 | -3.054836000 | 6.339867000 |
| 8 | 3.468375000 | -5.052522000 | -1.888921000 |
| 7 | 3.084022000 | -4.745490000 | -0.814327000 |
| 8 | 2.004219000 | -4.506267000 | -0.370588000 |

## [P$_{3333}$][Benzim] $NO_3$_NO

| | X | Y | Z |
|---|---|---|---|
| 15 | 0.188319000 | -1.995959000 | 1.423538000 |
| 6 | 3.991097000 | -0.708553000 | 2.584643000 |
| 1 | 3.793553000 | -0.502089000 | 3.640615000 |
| 1 | 4.524187000 | -1.657117000 | 2.513834000 |
| 6 | 2.696967000 | -0.777393000 | 1.775564000 |
| 1 | 2.192911000 | 0.197207000 | 1.790675000 |
| 1 | 2.944871000 | -0.993784000 | 0.733993000 |
| 1 | 4.644378000 | 0.079086000 | 2.201057000 |
| 6 | 1.766384000 | -1.873669000 | 2.314075000 |
| 1 | 2.243921000 | -2.850720000 | 2.246557000 |
| 1 | 1.527542000 | -1.712361000 | 3.368787000 |
| 6 | -0.700801000 | -3.511240000 | 1.901589000 |
| 1 | -1.420892000 | -3.723076000 | 1.105983000 |
| 1 | 0.046235000 | -4.306319000 | 1.871888000 |
| 6 | -1.411670000 | -3.456077000 | 3.259149000 |
| 1 | -2.171494000 | -2.667424000 | 3.254965000 |
| 1 | -0.692694000 | -3.197485000 | 4.040478000 |
| 6 | -2.063901000 | -4.797787000 | 3.593535000 |
| 1 | -1.310418000 | -5.585148000 | 3.644183000 |
| 1 | -2.804343000 | -5.074971000 | 2.836720000 |
| 1 | -2.578130000 | -4.751189000 | 4.557352000 |
| 6 | 0.411541000 | -1.927982000 | -0.381983000 |
| 1 | 0.951649000 | -0.999311000 | -0.586746000 |
| 1 | 1.077379000 | -2.744502000 | -0.668422000 |
| 6 | -0.901554000 | -1.972231000 | -1.168867000 |
| 1 | -1.580457000 | -1.154942000 | -0.911138000 |
| 1 | -1.431139000 | -2.915774000 | -1.024939000 |
| 1 | -0.680476000 | -1.890335000 | -2.234541000 |
| 6 | -0.827598000 | -0.520320000 | 1.844756000 |
| 1 | -0.537414000 | 0.262567000 | 1.138110000 |
| 1 | -1.866393000 | -0.770076000 | 1.612537000 |
| 6 | -0.698743000 | -0.005655000 | 3.281791000 |
| 1 | 0.316628000 | 0.331000000 | 3.497897000 |
| 1 | -0.964126000 | -0.764169000 | 4.018429000 |
| 1 | -1.365366000 | 0.846688000 | 3.429719000 |
| 8 | 4.294910000 | -7.165453000 | 1.363373000 |
| 7 | 4.483780000 | -6.309719000 | 2.181004000 |
| 8 | 2.017630000 | -4.483531000 | 0.633690000 |
| 7 | 3.347604000 | -5.972364000 | 2.904494000 |
| 6 | 3.320991000 | -4.968291000 | 3.871090000 |
| 6 | 1.991112000 | -4.963985000 | 4.334443000 |
| 7 | 1.232419000 | -5.917426000 | 3.636980000 |
| 6 | 2.050664000 | -6.473948000 | 2.796167000 |
| 1 | 1.812362000 | -7.237356000 | 2.074165000 |
| 6 | 4.286649000 | -4.092865000 | 4.344872000 |
| 1 | 5.292884000 | -4.093748000 | 3.947057000 |
| 6 | 3.883182000 | -3.202910000 | 5.338036000 |
| 1 | 4.601998000 | -2.495434000 | 5.736186000 |
| 6 | 2.569247000 | -3.200235000 | 5.833957000 |
| 1 | 2.302501000 | -2.502392000 | 6.620655000 |
| 6 | 1.607749000 | -4.079758000 | 5.340951000 |
| 1 | 0.596918000 | -4.092592000 | 5.731922000 |

| | | | | | | | |
|---|---|---|---|---|---|---|---|
| 8 | 4.055087000 | -3.787788000 | 0.965949000 | 8 | 3.092315000 | -3.458196000 | -0.959956000 |
| 7 | 3.075162000 | -3.916292000 | 0.199864000 | | | | |

## References


1. Greer, A. J., Taylor, S. F. R., Daly, H., Quesne, M., Catlow, C. R. A., Jacquemin, J. & Hardacre, C. Investigating the Effect of NO on the Capture of $CO_2$ Using Superbase Ionic Liquids for Flue Gas Applications. *ACS Sustain. Chem. Eng.* **2019**, *7*, 3567–3574, DOI 10.1021/acssuschemeng.8b05870.
